# Males Resemble Females: Re-Evaluating Sexual Dimorphism in *Protoceratops andrewsi* (Neoceratopsia, Protoceratopsidae)

**DOI:** 10.1371/journal.pone.0126464

**Published:** 2015-05-07

**Authors:** Leonardo Maiorino, Andrew A. Farke, Tassos Kotsakis, Paolo Piras

**Affiliations:** 1 Dipartimento di Scienze, Università Roma Tre, Rome, Italy; 2 Center for Evolutionary Ecology, Rome, Italy; 3 Raymond M. Alf Museum of Paleontology, Claremont, California, United States of America; NYIT College of Osteopathic Medicine, UNITED STATES

## Abstract

**Background:**

*Protoceratops andrewsi *(Neoceratopsia, Protoceratopsidae) is a well-known dinosaur from the Upper Cretaceous of Mongolia. Some previous workers hypothesized sexual dimorphism in the cranial shape of this taxon, using qualitative and quantitative observations. In particular, width and height of the frill as well as the development of a nasal horn have been hypothesized as potentially sexually dimorphic.

**Methodology/Principal Findings:**

Here, we reassess potential sexual dimorphism in skulls of *Protoceratops andrewsi *by applying two-dimensional geometric morphometrics to 29 skulls in lateral and dorsal views. Principal Component Analyses and nonparametric MANOVAs recover no clear separation between hypothetical “males” and “females” within the overall morphospace. Males and females thus possess similar overall cranial morphologies. No differences in size between “males” and “females” are recovered using nonparametric ANOVAs.

**Conclusions/Significance:**

Sexual dimorphism within *Protoceratops andrewsi *is not strongly supported by our results, as previously proposed by several authors. Anatomical traits such as height and width of the frill, and skull size thus may not be sexually dimorphic. Based on PCA for a data set focusing on the rostrum and associated ANOVA results, nasal horn height is the only feature with potential dimorphism. As a whole, most purported dimorphic variation is probably primarily the result of ontogenetic cranial shape changes as well as intraspecific cranial variation independent of sex.

## Introduction

Sexual dimorphism is an expected product of sexual selection and represents an important factor in breeding success within many species [1,2,3]. Morphology, behavior and size differences distinguish males and females in many extant and extinct animals [4–18]. However, recognizing sexual dimorphism in extinct organisms presents special problems in that it can be difficult to distinguish sexual differences from ontogenetic or intraspecific ones. This issue has plagued attempts to recognize sexual dimorphism in non-avian dinosaurs. For instance, hypothesized males and females of hadrosaurs were later shown to be stratigraphically and temporally separated species [19,20]. Purported differences between male and female tyrannosaurs in the number of chevron bones within the tail were recently attributed to interspecific variation independent of sex [21]. Recently, the finding of the medullary bone, typical of female birds, in the femur of a *Tyrannosaurus rex* individual has suggested that this investigated specimen was a female [22]. However, even in cases where sexual dimorphism seems plausible, limited sample sizes often prevent a rigorous test of the hypothesis.

The ceratopsians, or horned dinosaurs, are well represented in the fossil record by hundreds of well preserved skulls and lower jaws. They represent one of the best opportunities to infer sexual dimorphism within non-avian dinosaurs [23–35]. In the last decades, some of the more rigorous attempts, in many cases based on samples exceeding 10 or 20 individuals, hypothesized possible sexual dimorphism in the curvature and inclination of postorbital horns in chasmosaurines [27,28,36], in the display structures and cranial proportions of centrosaurines [37], and in the frill of protoceratopsids [23,25,38–40] (see Table 1). Conversely, more recent contributions highlighted how previously described sexual dimorphism within ceratopsids appears to be related to intra-specific and ontogenetic variation rather than sex-linked differences [32,33,41–43].

**Table 1:** List of neoceratopsian taxa for which sexual dimorphism has been previously proposed.

Taxon	Sexually dimorphic trait	Reference (dimorphism)	Reference (no dimorphism)
*Protoceratops andrewsi*	Nasal bump, height and width of the frill	[39]	[73, 74, this work]
*Protoceratops hellenikorhinus*	Nasal height, antorbital length and orientation of external nares	[31]	---
*Centrosaurus apertus*	Frill complexity and cranial robustness	[37]	[32, 43]
*Styracosaurus albertensis*	Nasal horn and frill complexity	[37]	[33, 41]
*Agujaceratops mariscalensis*	Curvature and inclination of postorbital horns	[27]	[3, 33]
*Anchiceratops ornatus*	Rostrum length and inclination of postorbital horns	[27]	[79]
*Chasmosaurus belli*	Inclination of postorbital horns	[27]	[34]
*Pentaceratops sternbergi*	Inclination of postorbital horns	[27]	[3, 33]
*Torosaurus latus*	Inclination of postorbital horns	[27]	[33]
*Triceratops horridus*	Inclination of postorbital horns	[27]	[42]

**Fig 1:**
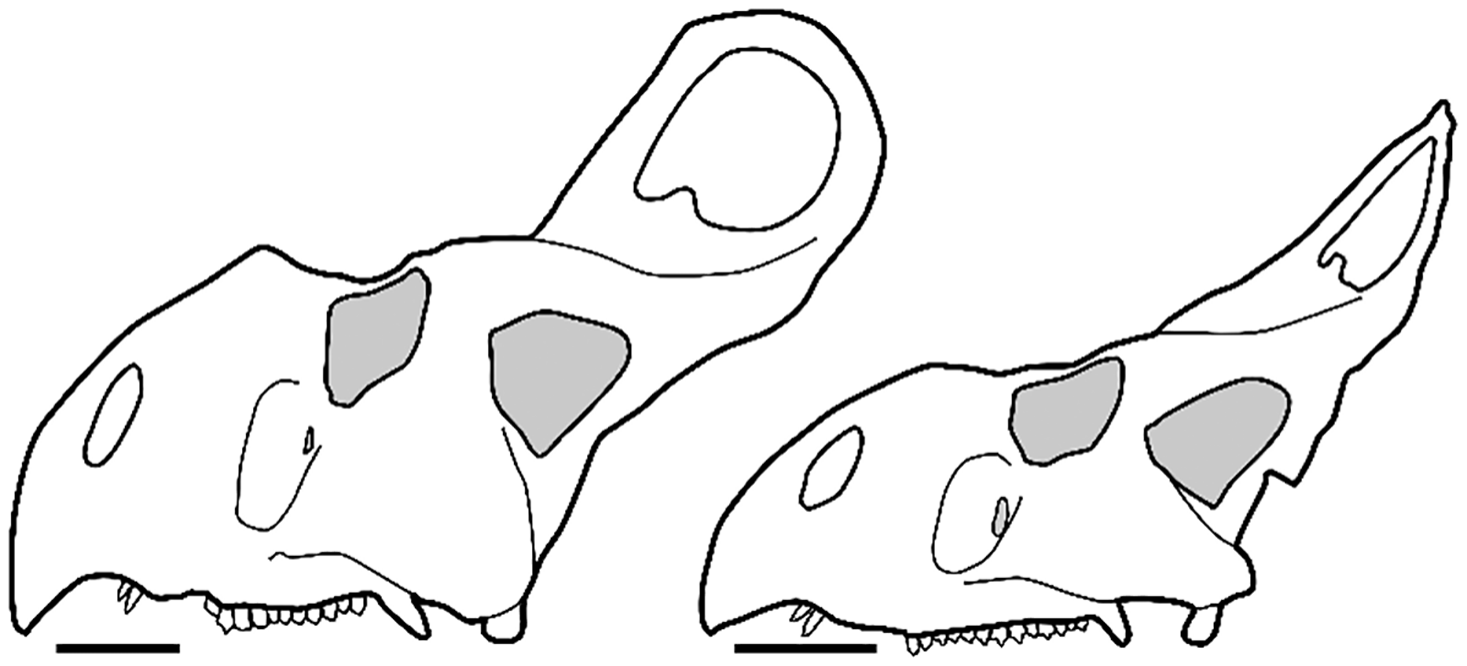
Hypothetical large “male” at left (AMNH 6438) and hypothetical large “female” at right (AMNH 6466). Redrawn and modified after Brown and Schlaikjer [23]. Scale equals to 10 cm.

*Protoceratops andrewsi* (Neoceratopsia, Protoceratopsidae) is a well-known neoceratopsian from the Campanian-aged Djadokhta Formation outcrops of the Gobi Desert, Mongolia [31]. It is a small (<2 m total body length), quadrupedal animal, characterized by a skull with a thin, bony frill projecting over the neck and lacking the prominent horns that characterized ceratopsids such as *Triceratops* (Fig 1). Beginning with the early Central Asiatic Expeditions of the American Museum of Natural History during the 1920s, more than one hundred well-preserved skulls and skeletons (many from various ontogenetic stages) have been unearthed, providing information on ontogeny and intraspecific variability within *P*. *andrewsi* [23,25,35,39,44].

Since the early scientific contributions on *Protoceratops*, several authors have hypothesized sexual dimorphism in cranial and postcranial features [23,39,40]. Dimorphism has been inferred for a variety of anatomical traits, such as the presence of a nasal horn and relative frill width, presumably used for visual display, ritualized combats and interspecific recognition [24,25,31,35]. Dodson [25] investigated allometric cranial shape changes in *Protoceratops* specimens (n = 24), using linear measurements of the skull analyzed with bivariate plots. Dodson hypothesized sexual dimorphism in the frill (width and height), postorbital width of the skull, and in the nasal horn (nasal height), suggesting that the frill had importance for visual display rather than simply being a structure for muscle attachments [26]. Recent descriptions of new specimens of *P*. *andrewsi* from Mongolia accepted this hypothesis and further highlighted inferred sexual dimorphism in the skull [35]. Fig 1 shows a hypothetical male and female of *P*. *andrewsi* following the work of Brown and Schlaikjer [23] and Dodson [25].

Dodson [25] specifically considered four variables as indicative of sexual dimorphism: (1) postorbital width of the skull, (2) nasal height of the skull, (3) width of the frill, and (4) height of the frill. Each variable for each specimen in the sample was assigned a score of -1 (male trait expression) or +1 (female trait expression) based on the specimen’s position relative to the line of best fit when each variable was regressed on basal skull length. A score of -4 indicates a “male” and a score of +4 indicates a “female” for all four characters. Only a few specimens reflect this assumption consistently across all characters; many specimens (e.g., AMNH 6439, AMNH 6425, AMNH 6438 and AMNH 6429) only partially correspond to “male” or “female” either. Dodson assigned the sex of these remaining specimens based on their position in the principal coordinates morphospace, suggesting *a posteriori* distinction for some “males” and “females” within *P*. *andrewsi*. However, no multivariate test has been performed to test for significantly different morphospace occupation between groups and thus to test the original hypothesis of sexual dimorphism within *P*. *andrewsi*.

Techniques such as geometric morphometrics (GM) in 2- or 3-dimensions, allow inference of patterns and processes during ontogeny, aspects of functional morphology and macroevolutionary patterns of phenotypes and allometric shape variation [45–61].

*Protoceratops andrewsi* is a suitable taxon for a GM study because it is represented by numerous well preserved skulls. In 1990, Chapman [38] applied a landmark-configuration technique to investigate qualitative shape changes in two skulls of *P*. *andrewsi* (a hypothetical male and hypothetical female) to re-evaluate and confirm the sexual dimorphism hypothesis. However, that was a preliminary study intended as an example of the use of geometric morphometrics, so the sample was very small, no statistical tests were undertaken, and overall results were presented in qualitative format [38]. In this work, we analyze phenotypic differences between 29 skulls, exploring cranial shape variation in *P*. *andrewsi* using a landmark configuration on skulls and statistical tools, in order to more thoroughly assess the hypothesis of cranial sexual dimorphism.

## Materials and Methods

### Material

Institutional abbreviations: **AMNH**, American Museum of Natural History, New York, NY, U.S.A.; **CM**, Carnegie Museum of Natural History, Pittsburgh, PA, U.S.A.; **DMNH**, Denver Museum of Nature and Science, Denver, CO, U.S.A.; **MPC**, Mongolian Paleontological Collection, Ulan Bataar, Mongolia.; **UALVP**, University of Alberta, Laboratory of Vertebrate Paleontology, Edmonton, Canada.

We collected photos of 29 skulls in dorsal and lateral view (including juveniles, subadults and adults) of *P*. *andrewsi* (Table A in S1 File). A Canon 400D camera was used to collect most pictures, and where necessary additional images were taken from the literature. We followed the protocols of Marcus et al. [62] and Mullin and Taylor [63] to minimize parallax and measurement error in the photographs. We obtained permission from the relevant museums or institutions to access the collections for photography.

### Methods

#### Sex Attribution

**Fig 2:**
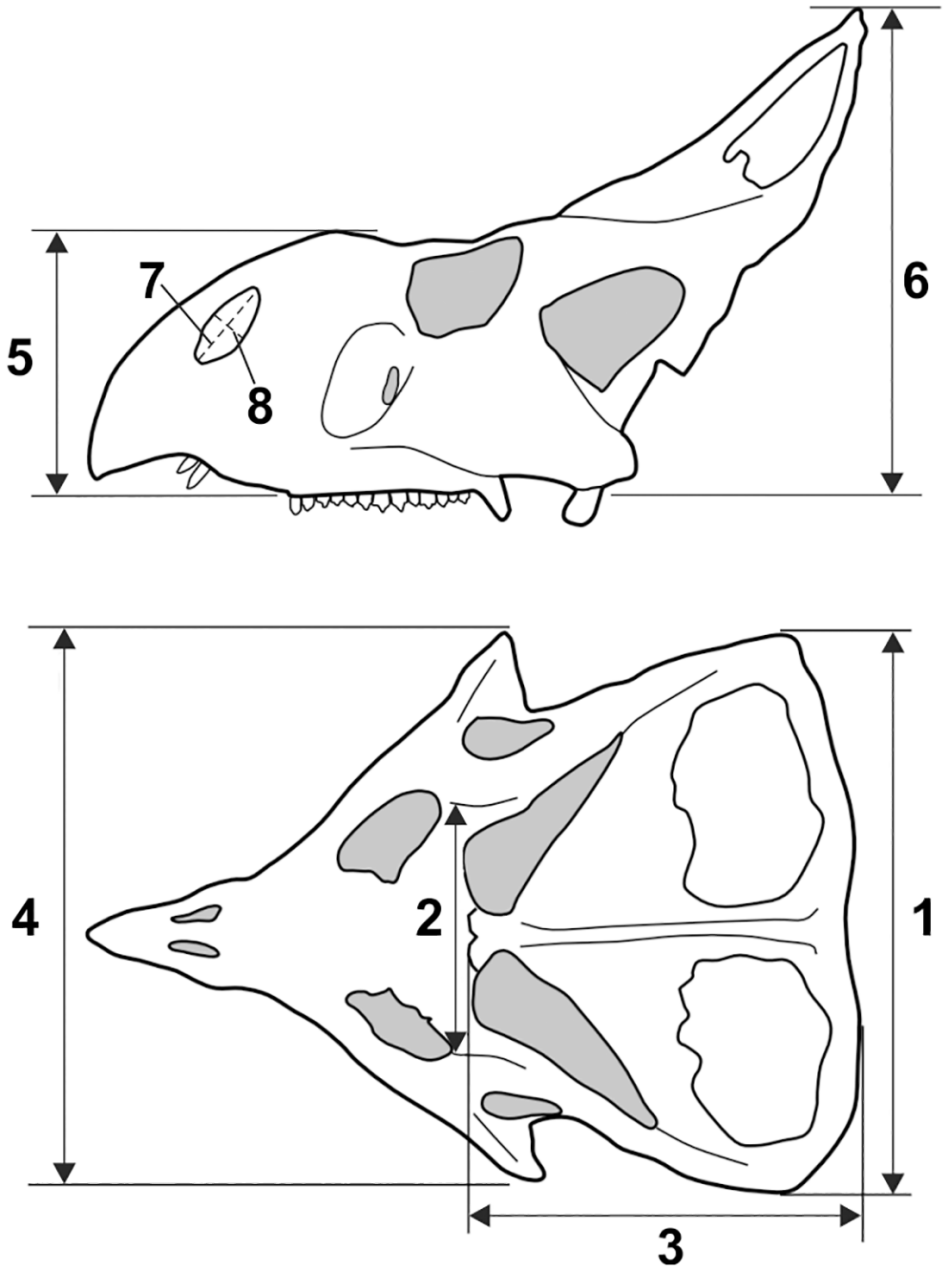
Linear measurements measured on skull. Redrawn following the measurement standards originally in Dodson [25]. The numbers of measurements corresponds to those reported in Table B in S1 File.

**Fig 3:**
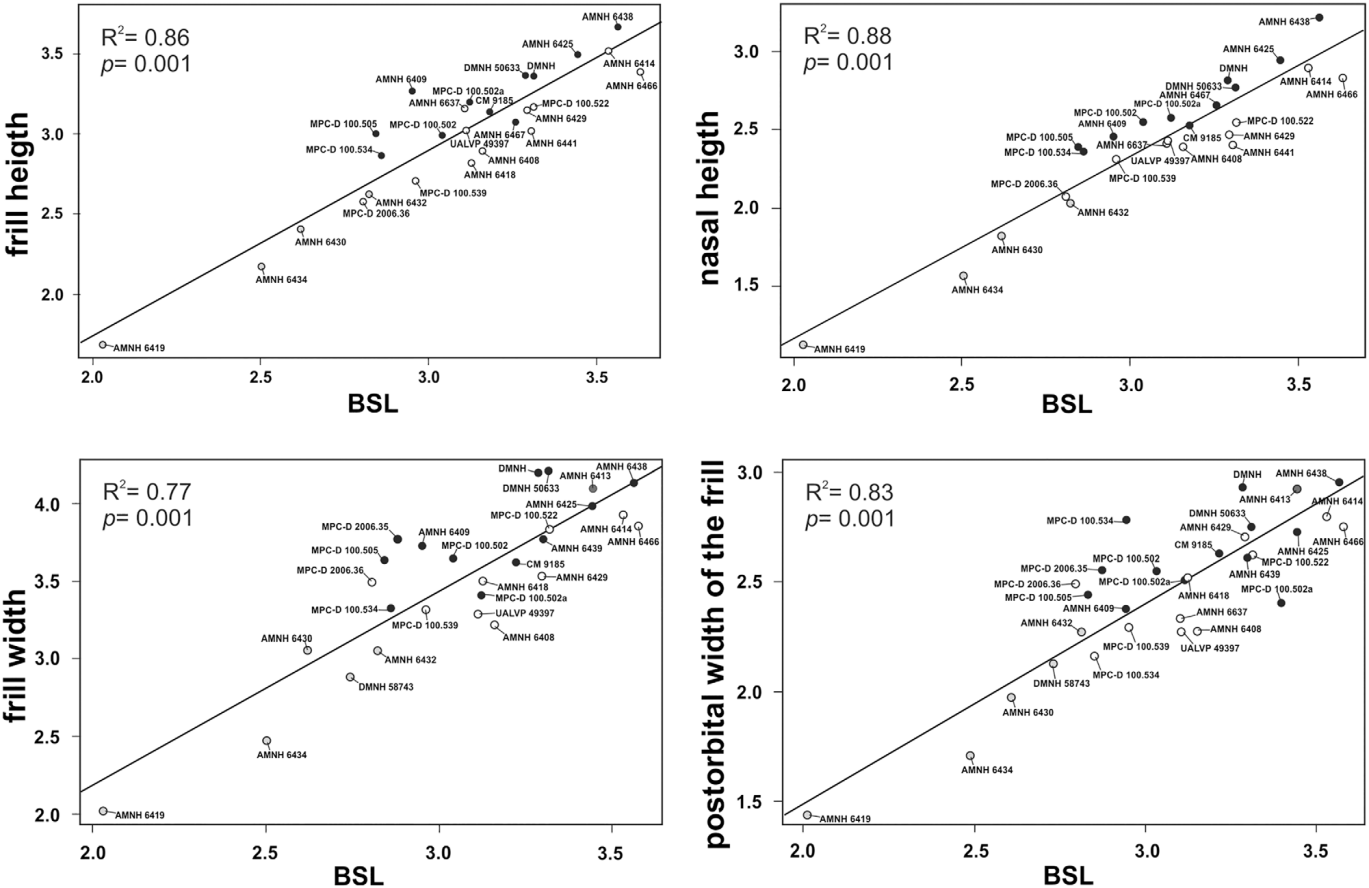
Linear regressions of basal skull length (BSL) against the four sex-discriminant variables of the *Protoceratops* sample (see Table B in S1 File). Black dot represents “male”, white dot represents “female”, gray dot represents juvenile, and red dot represents a sex-undetermined specimen. Scale of axes is logarithmic.

According to previous works [25], the most critical anatomical features for identifying sexual dimorphism within *P*. *andrewsi* are the postorbital width of the skull, width of the frill, nasal height of the skull, height of the frill, and length and width of external nares. We took linear measurements from photographs of skulls (dorsal and lateral view) (Fig 2) using tpsDig2 v2.16 [64] in order to distinguish *a priori* “males” and “females” in the sample, according to the criteria specified in Dodson [25] (Table B in S1 File). Although direct measurements would of course be most desirable, they were not possible in many cases due to specimen access constraints (e.g., unremovable display cases). We measured basal skull length (BLS) from photos of the sex-undetermined specimens (unshared with Dodson’s work) and we regressed BSL with each of the sex-discriminant traits, as indicated by Dodson [25]. Specimens which lie below the line of best fit have a score of +1 (female trait) and specimens which lie above the line have a score of -1 (male trait; Table 2 and Fig 3). We note that in all cases, Dodson’s original sex determinations are confirmed here for specimens in his sample.

**Table 2:** Sexual scores for specimens of *P*. *andrewsi* unshared with Dodson’s work [25].

Specimen #	Postorbital width	Frill width	Nasal height	Frill height	Total score
DMNH no code	-1	-1	-1	-1	-4
DMNH 50633L	-1	-1	-1	-1	-4
DMNH 58743	+1	+1	NA	NA	+2
AMNH 6418	+1	-	NA	+1	+2
AMNH 6637	+1	NA	+1	-1	+1
UALVP 49397	+1	+1	+1	-	+3
CM 9185	-1	+1	-	-1	-1
MPC-D 100.502	-1	-1	-1	-1	-4
MPC-D 100.502a	-	+1	-1	-1	-1
MPC-D 100.505	-1	-1	-1	-1	-4
MPC-D 2006.36	-1	-1	+1	+1	0
MPC-D 100.522	+1	-	+1	+1	+3
MPC-D 100.534	1	-1	-1	-1	-2
MPC-D 100.539	+1	+1	-1	+1	+2
MPC-D 2006.35	-1	-1	NA	NA	-2

NA = not available. DMNH 58743 represents a juvenile.

#### Geometric Morphometrics

**Fig 4:**
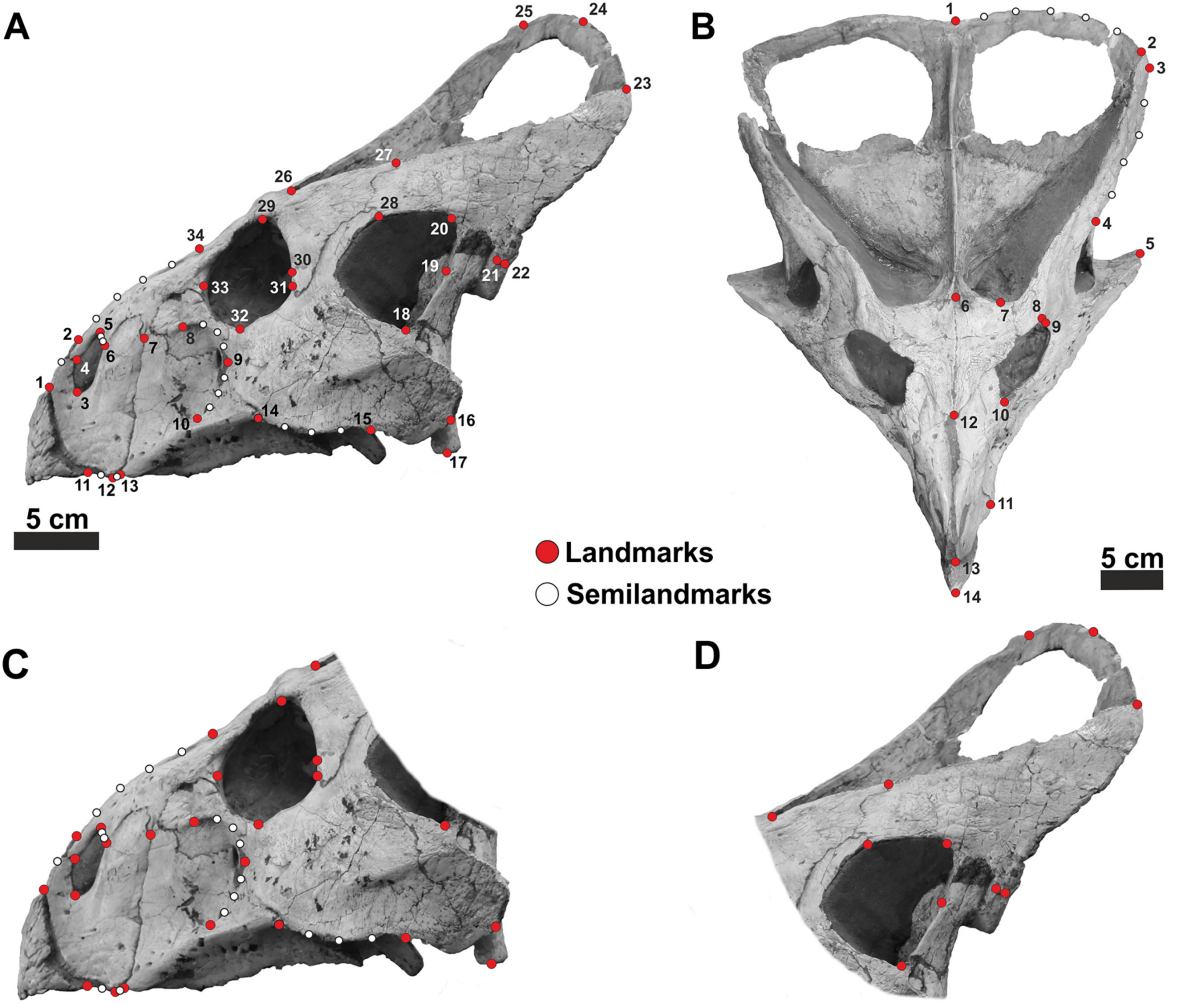
Landmark and semi-landmark configurations. A, landmark configuration for skull in lateral view. B, landmark configuration for skull in dorsal view. C and D are subunits of the skull configuration. Landmarks have identical definitions. See Table C in S1 File for landmark definitions.

Geometric morphometrics (GM) is suitable to quantify morphological changes and to analyze phenotypic differences among taxa [48,60]. Thirty-four landmarks and 18 semilandmarks in two dimensions were digitized on each skull in lateral view (Fig 4A), and 14 landmarks and 9 semilandmarks were digitized on each skull in dorsal view (Fig 4B) using tpsDig2 v2.16 software [64]. Landmarks were selected to identify major sutural contacts on the skull (Table C in S1 File), as well as to capture the shapes of structures previously hypothesized to be sexually dimorphic. Scale bars were used to scale each digitized specimen.

Taphonomic distortion represents a major concern in a GM study focusing on fossil taxa, because deformation or damage obscures the original cranial shape and thus may potentially affect the results. Although no method will completely remove the effects of distortion, several strategies were used to reduce the effects within our sample. For analyses of skulls in dorsal view, we only digitized the half of the skull that was least affected by distortion.

We removed from the sample specimens such as AMNH 6443, AMNH 6428, AMNH 6477, AMNH 6417 or AMNH 6251 (holotype), because they were too strongly distorted, damaged, or had major anatomical parts restored with plaster. By contrast, some specimens presented what we consider a reasonable and minor amount of restoration. For instance, AMNH 6438, AMNH 6432, AMNH 6637 and AMNH 6430 have partially restored frills. The parietals are lost in MPC-D-100.539, but the rest of the skull is preserved in lateral view. Thus, we included these specimens in the analysis to maximize sample size. We used the function fixLMtps() from the “Morpho” R package [65] to estimate missing landmarks based on the three closest complete specimens to minimize errors during the digitization process that result from restoration.

Even if the use of 2D representations loses some data relative to 3D geometries [66], we feel that a 2D approach is justified here due to inaccessibility of many specimens within museum exhibits. For instance, many specimens currently are obscured by mounting hardware or glass exhibit cases. Furthermore, based on the criteria presented by Dodson [25], we posit that sexual dimorphism can be assessed with a 2D approach.

Semilandmarks are useful to capture morphological information of outlines where no homologous points can be detected. Curve or contours are assumed to be homologous among specimens [67,68]. We digitized semilandmarks at equal distance along outlines drawn on the specimens. Moreover, we digitized skull photos considering two separate subsets of landmarks. We also explored the cranial shape changes focusing on the skull without the frill and the frill alone (in lateral view) to investigate if either module possesses discriminating anatomical traits [46] (Fig 4C and 4D).

Generalized Procrustes Analysis (GPA, [69]) implemented using the procSym() function in the “Morpho” R package [65] was used to analyze shape differences among specimens in the four different samples (i.e. entire skull, in lateral and dorsal view, skull without the frill and the frill alone, in lateral view). GPA scales, aligns and rotates each landmark configuration to unit centroid size (CS = the square root of sum of squared differences between landmarks from their centroid [70]). CS represents a proxy for size of the *Protoceratops* specimens in our sample [66].

In the sample used here, a few specimens (AMNH 6418, AMNH 6637 and AMNH 6413) exhibit ambiguous characters for *a priori* distinction of “males” and “females”. However, after changing the sex attribution of these specimens, the overall results of the analyses were unchanged. In addition, we provide the raw TPS files (S2 File), which will allow future researchers to reanalyze the data in light of our findings.

After GPA, Principal Components Analysis (PCA) was performed on the Procrustes coordinates to identify orthogonal axes of maximal variation in the four datasets. Additionally, we performed a PCA on linear measurements calculated on the skulls, in dorsal and lateral view, to test if the anatomical traits (e.g. nasal height of skull and width of the frill) suggested by Dodson [25] as critical for sexual dimorphism in *P*. *andrewsi*, are useful to distinguish “males” and “females”.

#### Linear Models and UPGMA

Overall nonparametric permuted MANOVA and ANOVA (npMANOVA and npANOVA; 10,000 permutations) have been performed on the four pooled datasets to highlight difference in shape and size, respectively. Pair-wise npMANOVA (using sex as a factor), and corrected after Holm correction, was performed on all shape variables as well as on the linear measurements in order to test for sexual dimorphism among specimens of *P*. *andrewsi* in the four datasets. Additionally, a pair-wise npANOVA (using sex as a factor), and corrected after Holm correction, was performed to explore differences in size (measured as CS). We performed this tests using the function adonis() of the “vegan” R package [71] that manages with unbalanced sample size.

Due to the small sample available, no linear discriminant analysis has been performed. As stated in a recent paper [72], when the number of variables is close to the number of cases, the groups appear separated after performing a linear discriminant analysis or a canonical variate analysis even if the individuals come from the same population. Therefore, a valid assessment of group separation needs many more cases than variables [72].

A npANOVA (using sex as a factor, and a Holm correction for *p*-values) was performed to explore differences between each PC score accounting for up to 5% of shape variation.

Additionally, a cluster analysis was performed on the shape data of the four datasets. Procrustes distances were agglomerated by means of a UPGMA (Unweighted Pair Group Method with Arithmetic mean) algorithm. The results are four dendrograms of morphological similarities among specimens included in the sample (skull in lateral and dorsal view, skull without frill and sole frill).

Lastly, in order to explore ontogenetic shape changes in the skull, we examined the relationship between shape (as dependent) and size (as independent) variables, in the cranial datasets in lateral and dorsal view, by using a Ordinary Least Squares (OLS) linear regression model. Because we do not have any histological data for the studied specimens to assess the age at death of each, we used size as proxy for age [66]. Just for sake of visualization, we used Canonical Correlation Analysis (CCA) in order to extract the vector maximally correlated with the independent variable (size).

## Results

First, npMANOVAs performed on the four pooled samples highlight difference in shape within each dataset (*p*-value <<0.05), whereas npANOVAs show significant differences in size (*p*-value <<0.05).

**Fig 5:**
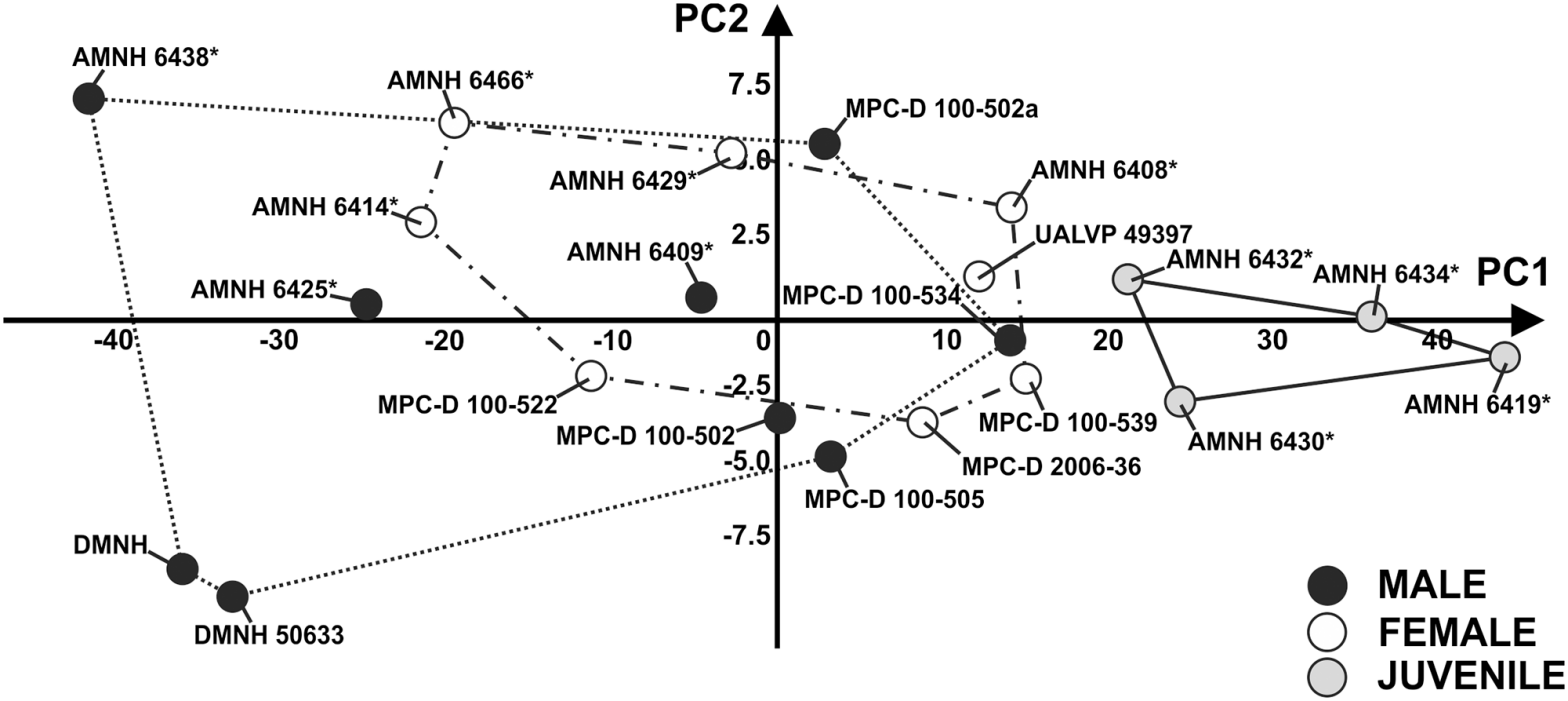
Principal Component Analysis performed on the linear measurements of skulls. The continuous line represents “juvenile” morphospace. The dotted line represents “male” morphospace and the dashed line represents “female” morphospace. Asterisks indicate specimens shared with Dodson [25].

We performed a PCA on linear skull measurements (dorsal and lateral view) to test the hypothesis of sexual dimorphism within *P*. *andrewsi*. The first 3 PCs explains collectively 98.7% of total shape variance. Fig 5 shows the relationship between PC1 (93.19% of shape variance) and PC2 (3.44% of shape variance) for linear measurements measured on the skull. At positive PC1 values, juveniles cluster together. On the other hand, “males” and “females” do not differ from each other at negative PC1 values or positive and negative PC2 values.

**Fig 6:**
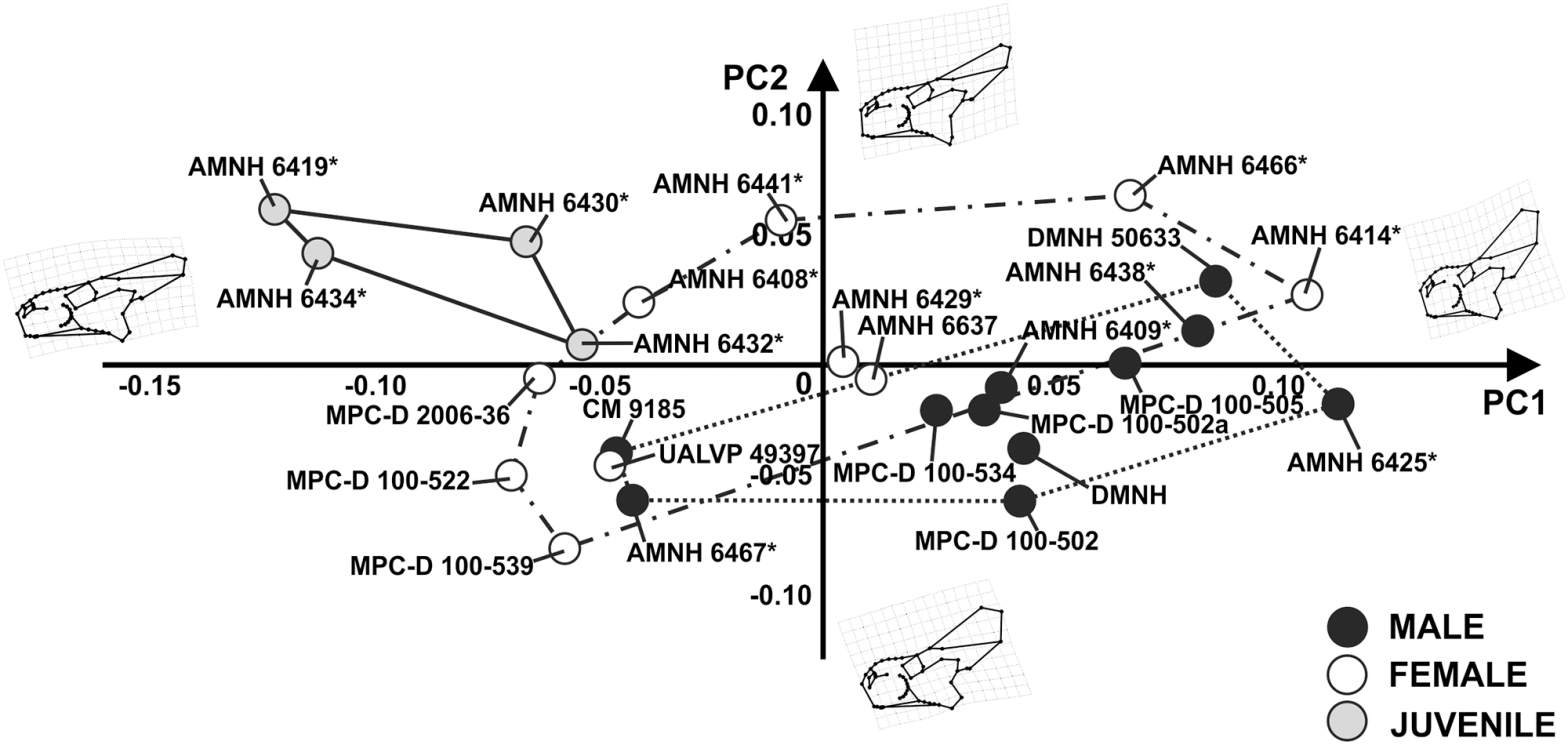
Principal Component Analysis performed on the skulls in lateral view. The continuous line represents “juvenile” morphospace. The dotted line represents “male” morphospace and the dashed line represents “female” morphospace. Asterisks indicate specimens shared with Dodson [25].

The first 15 principal components of PCA explain 95% of total shape variance of skulls in lateral view. Fig 6 shows the relationship between PC1 (34.74% of the total shape variance) and PC2 (12.78% of the total shape variance) of the total sample. Positive PC1 values are associated with a massive skull bearing a high and dorsally expanded frill, small orbit (relative to the rest of the skull), elevated snout, pronounced nasal horn and a ventrocaudally expanded jugal. This morphology is typical of an adult. At negative PC1 values, the skull is short and narrow with a short frill, no nasal horn, large orbit (relative to the rest of the skull) and a premaxilla-maxilla upper contact rising to the same height as the lower margin of the orbit. This morphology corresponds to a juvenile. At positive PC2 values, the skull has a caudodorsally elongated squamosal, a ventrally expanded jugal, a less pronounced nasal horn and a caudally tilted external naris. At negative PC2 values the skull bears a caudally expanded jugal, a dorsally expanded parietal, a pronounced nasal horn and a nearly vertical external naris. Juveniles cluster at negative PC1 values, whereas “male” and “female” morphospace overlaps at negative and positive PC1 and PC2 values, indicating morphological similarities between groups.

S1 Fig shows the relationship between PC1, PC2 and PC3 (the latter corresponding to 10.57% of the total shape variance), and Table D in S1 File reports the shape variance explained by each of first 15 Principal Components.

**Fig 7:**
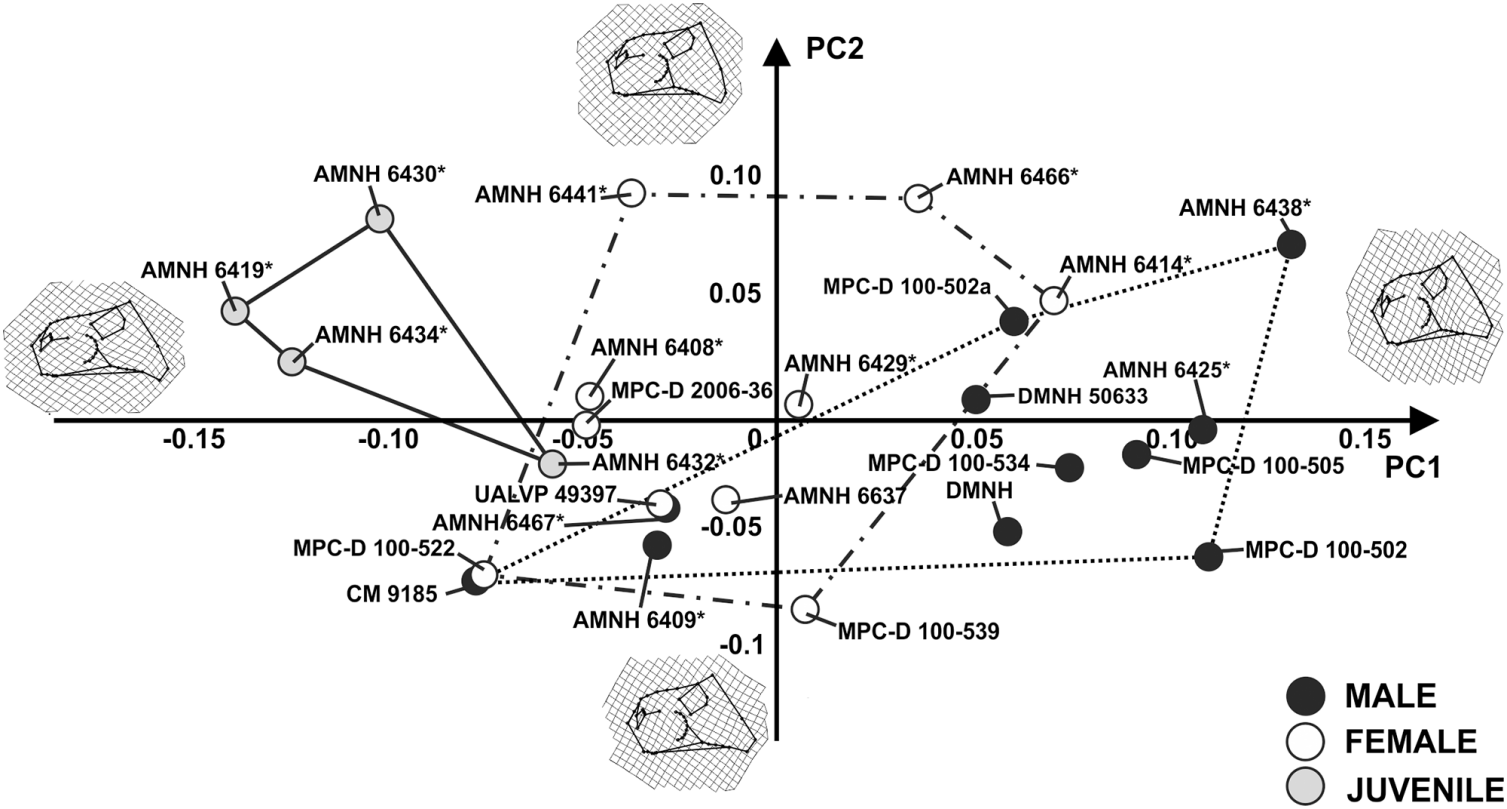
Principal Component Analysis performed on skulls with the frill excluded. The continuous line represents “juvenile” morphospace. The dotted line represents “male” morphospace and the dashed line represents “female” morphospace. Asterisks indicate specimens shared with Dodson [25].

The first 13 principal components of PCA, performed on lateral views of skulls with the frill excluded, explain collectively 95% of total shape variance. Fig 7 shows the relationship between PC1 (33.93% of shape variance) and PC2 (16.23% of shape variance) of the total sample. A *Protoceratops* adult cranial morphology having a massive skull with a high snout, pronounced nasal horn and a caudoventrally expanded jugal is associated with positive PC1 values. A short skull possessing a short snout, no nasal horn, large orbit and a narrow, caudally expanded jugal characterizes *Protoceratops* juvenile cranial morphology, at negative PC1 values. At negative PC2 values, the skull is short with a nearly vertical external naris, a dorsally place maxilla-premaxilla contact, and a caudally expanded jugal. At positive PC2 values, the skull is relatively longer, bearing an external naris tilted backwards and a maxilla-premaxilla upper contact rising to the same height as the lower margin of the orbit. Juveniles are distinct from “males” and “females” at negative PC1 values, whereas “male” and “female” morphospaces overlap each other.

**Fig 8:**
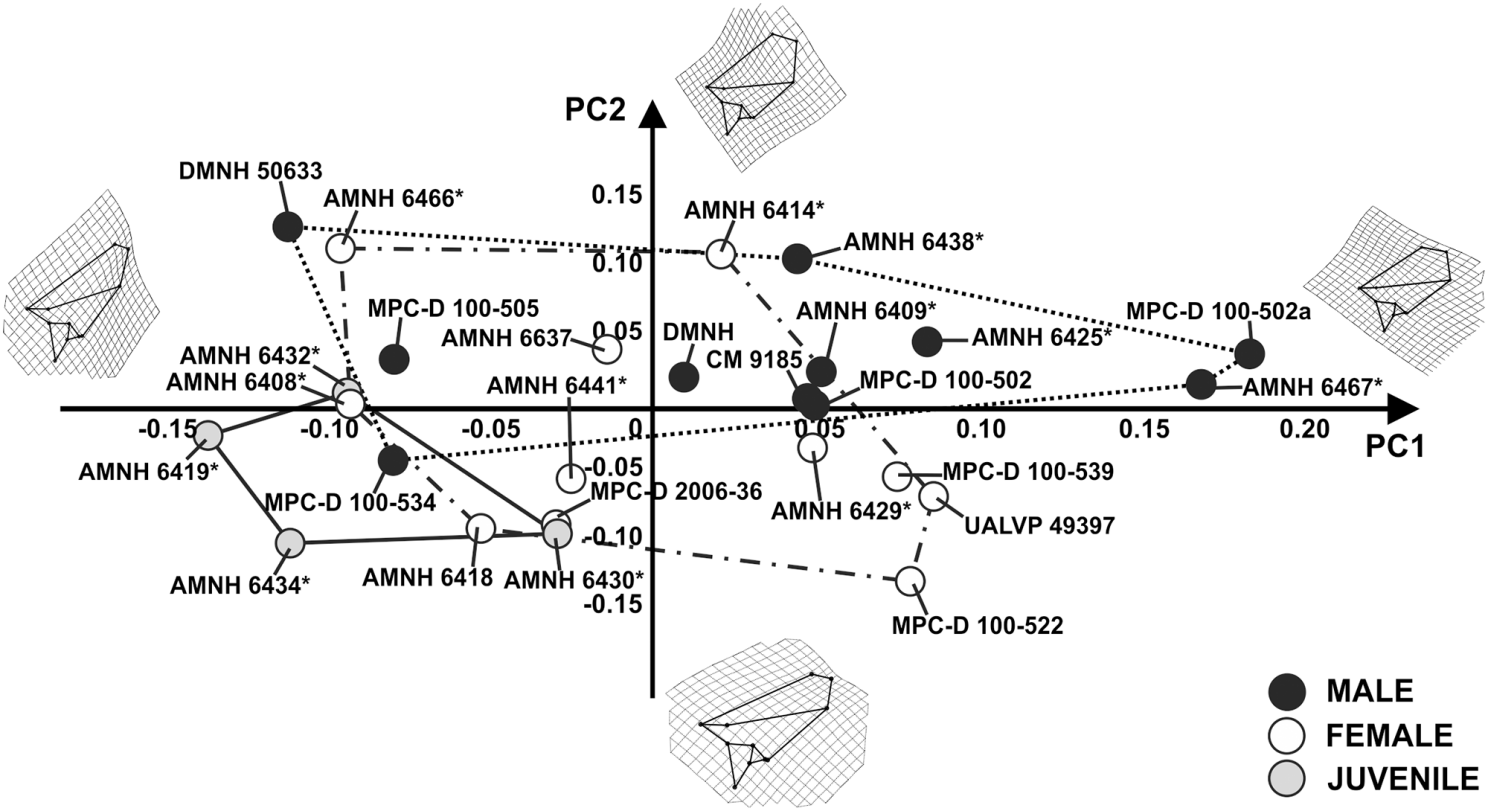
Principal Component Analysis performed on the frills. The continuous line represents “juvenile” morphospace. The dotted line represents “male” morphospace and the dashed line represents “female” morphospace. Asterisks indicate specimens shared with Dodson [25].

The first 9 principal components of PCA, performed on GM data of the frill in lateral view, explain collectively 95% of total shape variance. Fig 8 shows the relationship between PC1 (32.05% of shape variance) and PC2 (21.04% of shape variance) of the total sample. A frill bearing a dorsally expanded parietal and a caudally elongated squamosal is associated with positive PC1 values. A shorter frill with a dorsally expanded parietal-squamosal complex is associated with negative PC1 values. At positive PC2 values the frill possesses a dorsally expanded parietal and a large squamosal, whereas at negative PC2 values the frill bears a longer squamosal and a caudodorsally expanded parietal. Juvenile, “male” and “female” morphospaces greatly overlap each other.

**Fig 9:**
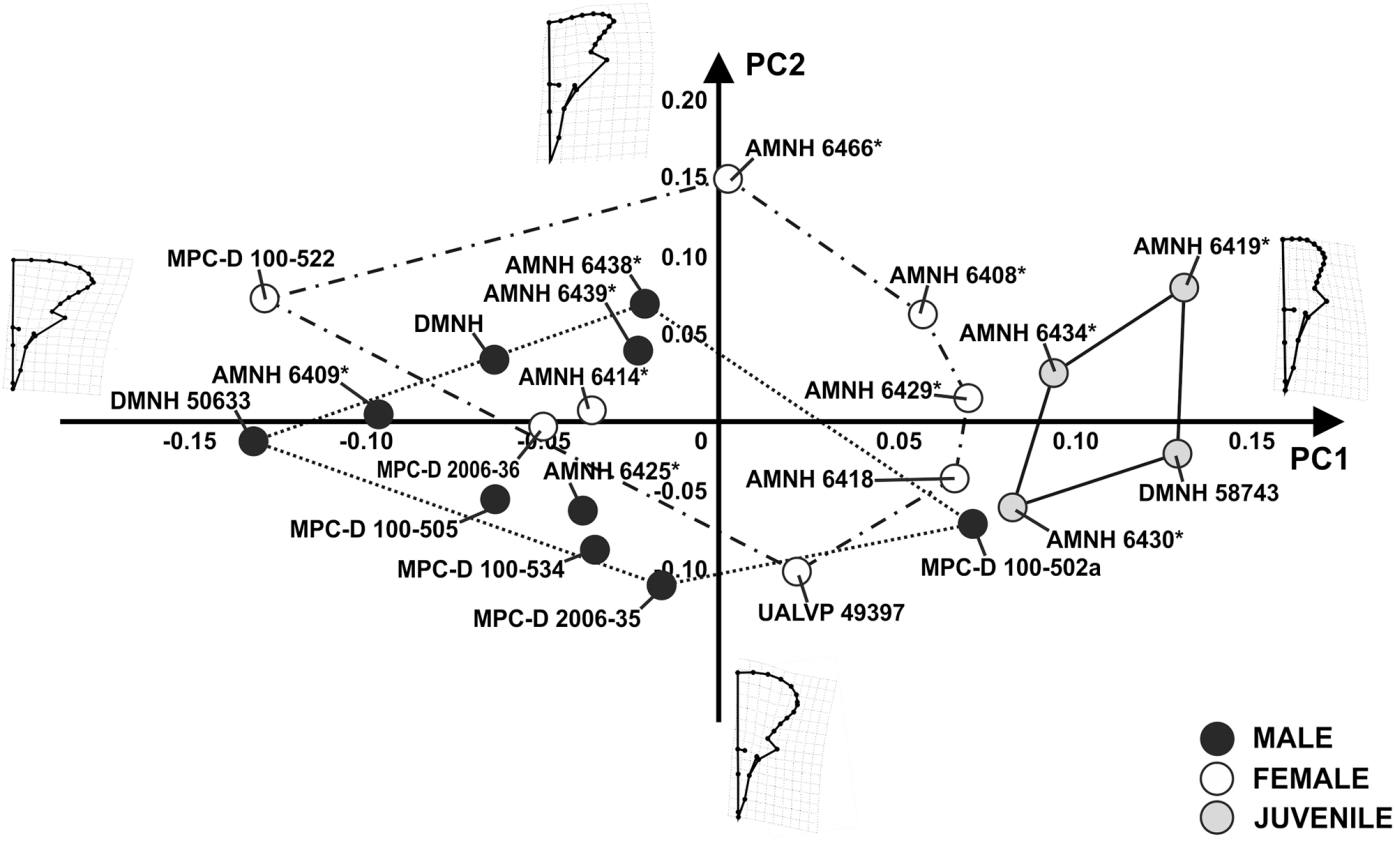
Principal Component Analysis performed on skulls in dorsal view. The continuous line represents “juvenile” morphospace. The dotted line represents “male” morphospace and the dashed line represents “female” morphospace. Asterisks indicate specimens shared with Dodson [25].

The first 8 principal components of the PCA, performed on the skulls in dorsal view, explain collectively 96% of total shape variance. Fig 9 shows the relationship between PC1 (41.36% of shape variance) and PC2 (30.54% of shape variance) of the total sample. At negative PC1 values the skull possesses a broad and laterally expanded frill, small orbit and a caudolaterally expanded jugal. This cranial morphology corresponds to a *Protoceratops* adult. At positive PC1 values the skull has a short and caudally expanded frill, a large orbit and a moderately laterally expanded jugal. This morphology corresponds to a *Protoceratops* juvenile. At positive PC2 values the skull bears a short and caudolaterally expanded frill, long snout and a jugal that is deeply expanded caudolaterally. A skull with a wide and caudally expanded frill and a shorter snout is associated with negative PC2 values. Juvenile, “male” and “female” morphospaces still greatly overlap.

S2 Fig shows the relationship between PC1, PC2 and PC3 (the latter explains 10.40% of the total shape variance), and Table E in S1 File reports the shape variation explained by each of the first 8 Principal Components.

The pair-wise npMANOVAs performed on the linear measurements and Procrustes shape variables between groups (i.e. sex; Table 3) did not highlight any significant morphological differences between “males” and “females” of *P*. *andrewsi*. After a Holm correction, only the skull shape with the frill excluded appears to differentiate males and females. Both of them are separated from juveniles.

**Table 3:** Pair-wise npMANOVA performed on the shape variables per-group.

	Male	Female	Juvenile
Linear measurements on skulls			
Male (n = 8)	---	0.24	**0.004**
Female (n = 8)	0.23	---	**0.008**
Juvenile (n = 4)	**0.01**	**0.015**	---
Skull (lateral view)			
Male (n = 11)	---	0.062	**0.009**
Female (n = 10)	0.061	---	**0.01**
Juvenile (n = 4)	**0.003**	**0.02**	---
Skull without frill (lateral view)			
Male (n = 11)	---	0.052	**0.008**
Female (n = 10)	**0.049**	---	**0.0085**
Juvenile (n = 4)	**0.001**	**0.013**	---
Frill (lateral view)			
Male (n = 11)	---	0.11	**0.003**
Female (n = 11)	0.108	---	**0.016**
Juvenile (n = 4)	**0.008**	**0.027**	---
Skull (dorsal view)			
Male (n = 10)	---	0.203	**0.001**
Female (n = 8)	0.196	---	**0.0.18**
Juvenile (n = 4)	**0.002**	**0.044**	---

Statistically significant results (*p*<0.05) are indicated in bold. Statistically significant results (*p*<0.05) after a Holm correction are shown in the lower left triangle.

Pair-wise npANOVAs show a clear difference in size between juveniles and adults but not between “males” and “females” (which include adults and sub-adults; Table 4) in the datasets. After a Holm correction, pair-wise ANOVAs have similar results.

**Table 4:** Pair-wise npANOVA performed on the size variables (CS) per-group.

	Male	Female	Juvenile
Skull (lateral view)			
Male (n = 11)	---	0.801	**0.001**
Female (n = 10)	0.81	---	**0.001**
Juvenile (n = 4)	**0.003**	**0.003**	---
Skull without frill (lateral view)			
Male (n = 11)	---	0.90	**0.0006**
Female (n = 10)	0.91	---	**0.0019**
Juvenile (n = 4)	**0.003**	**0.003**	---
Frill (lateral view)			
Male (n = 11)	---	0.32	**0.0011**
Female (n = 11)	0.32	---	**0.005**
Juvenile (n = 4)	**0.0012**	**0.01**	---
Skull (dorsal view)			
Male (n = 10)	---	0.95	**0.0056**
Female (n = 8)	0.93	---	**0.0042**
Juvenile (n = 4)	**0.013**	**0.013**	---

Statistically significant results (*p*<0.05) are indicated in bold. Statistically significant results (*p*<0.05) after a Holm correction are shown in the lower left triangle.

Pair-wise npANOVAs were performed on each PC score that accounts for up to 5% of shape variation, highlighting a non-significant difference between “male” and “female” cranial shape associated with each PC (Table F in S1 File). The only exception is represented by PC1 of the skulls exclusive of the frill (Table 5). Thus, these results seem to identify shape differences between groups.

**Table 5:** Pair-wise npANOVA performed on each PCscore variable per-group.

	Male	Female	Juvenile
Skull without frill (lateral view)—PC1(33.9%)			
Male (n = 11)	---	**0.023**	**0.001**
Female (n = 10)	**0.024**	---	**0.023**
Juvenile (n = 4)	**0.003**	**0.007**	---
Skull without frill (lateral view)—PC2(16.2%)			
Male (n = 11)	**---**	0.39	0.07
Female (n = 10)	0.78	**---**	0.39
Juvenile (n = 4)	0.21	0.78	---
Skull without frill (lateral view)—PC3(9.5%)			
Male (n = 11)	---	0.59	0.59
Female (n = 10)	1	**---**	0.38
Juvenile (n = 4)	1	1	---
Skull without frill (lateral view)—PC4(9.08%)			
Male (n = 11)	**---**	0.89	0.26
Female (n = 10)	0.9	**---**	0.22
Juvenile (n = 4)	0.64	0.64	---
Skull without frill (lateral view)—PC5(5.7%)			
Male (n = 11)	**---**	0.73	0.69
Female (n = 10)	1	**---**	0.82
Juvenile (n = 4)	1	1	---
Skull without frill (lateral view)—PC6(4.7%)			
Male (n = 11)	**---**	0.98	0.81
Female (n = 10)	1	**---**	0.81
Juvenile (n = 4)	1	1	---

Statistically significant results (*p*<0.05) are indicated in bold. Statistically significant results (*p*<0.05) after a Holm correction are shown in the lower left triangle.

**Fig 10:**
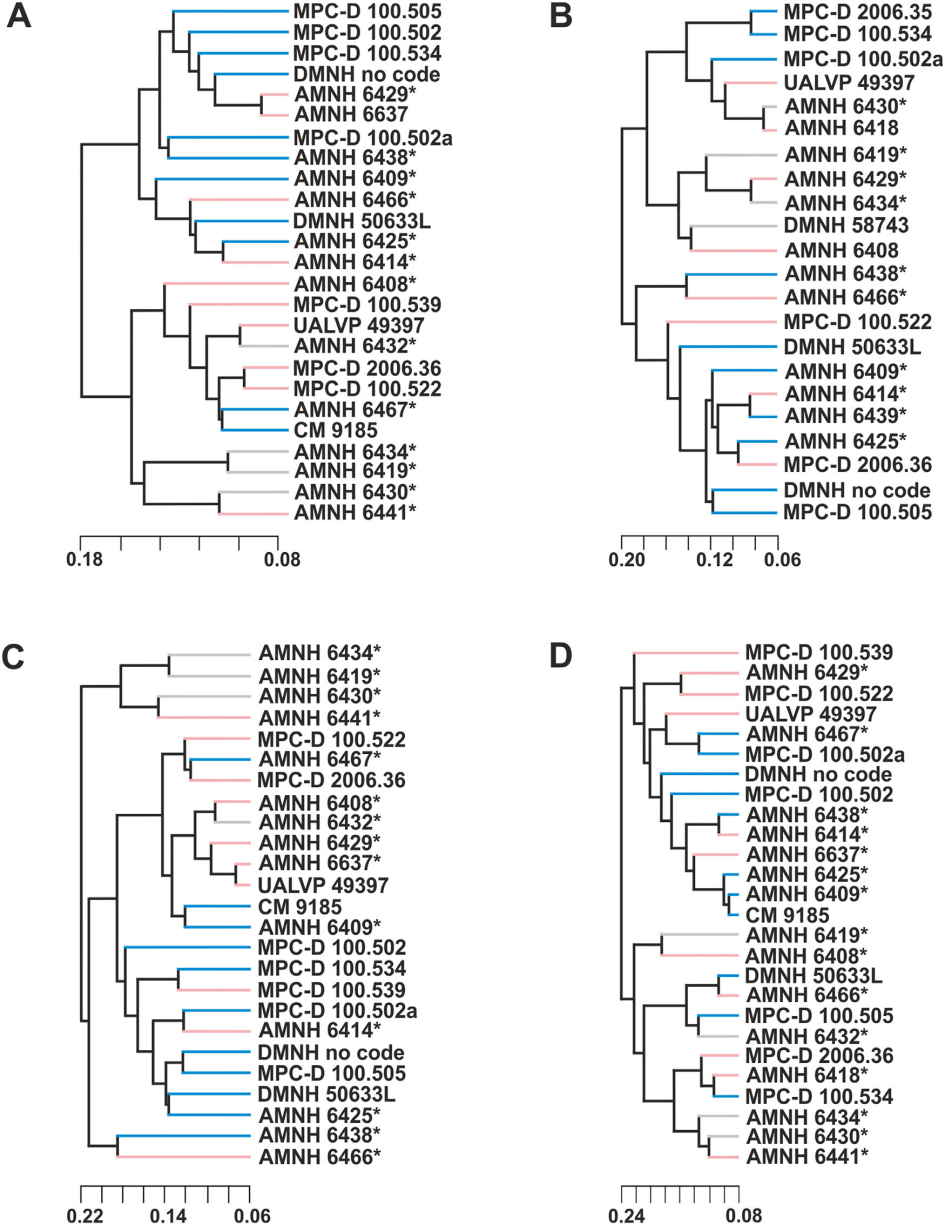
UPGMA cluster analysis performed on the four samples. A, UPGMA cluster analysis of skulls in lateral view. B, UPGMA cluster analysis performed on skulls in dorsal view. C, UPGMA cluster analysis of skulls without frill. D, UPGMA cluster analysis performed on the frills. Asterisks indicate specimens shared with Dodson [25]. Light blue indicates a “male”, pink indicates a “female”, grey indicates a juvenile.

The UPGMA dendrograms of morphological similarities of entire skull shape in lateral and dorsal view (Fig 10A and 10B) highlight no obvious morphological differences between “males” and “females”. Juveniles resemble the “female” cranial shape.

Cluster analysis performed on the sample of skulls excluding the frill (Fig 10C) also shows no clear separations between “males” and “females”, nor does it show other groupings that might suggest alternative groupings for sexual dimorphism. Even if the ANOVA itself seems to identify two groups, some “males” have similar rostrum shape to the “females” and vice versa. Juveniles are distinguished from “female” rostrum shape. In the UPGMA dendrogram of frill shape similarities (Fig 10D), “males”, “females” and juveniles do not show any clear separation on the basis of morphological differences.

### Ontogenetic shape changes

Regressing shape and size documents the ontogenetic changes in the cranial sample of *P*. *andrewsi* in lateral and dorsal view. The OLS results highlights a significant relationship between shape and size (cranial sample in lateral view, R^2^: 0.226, *p*-values: 0.001; cranial sample in dorsal view, R^2^: 0.126, *p*-values: 0.016).

At small size values of skulls, in lateral view, *P*. *andrewsi* possesses a large orbit, short rostrum, slender and small squamosal, a low frill and a nasal with no horn. This morphology corresponds to a juvenile, as noted in previous studies [23].

**Fig 11:**
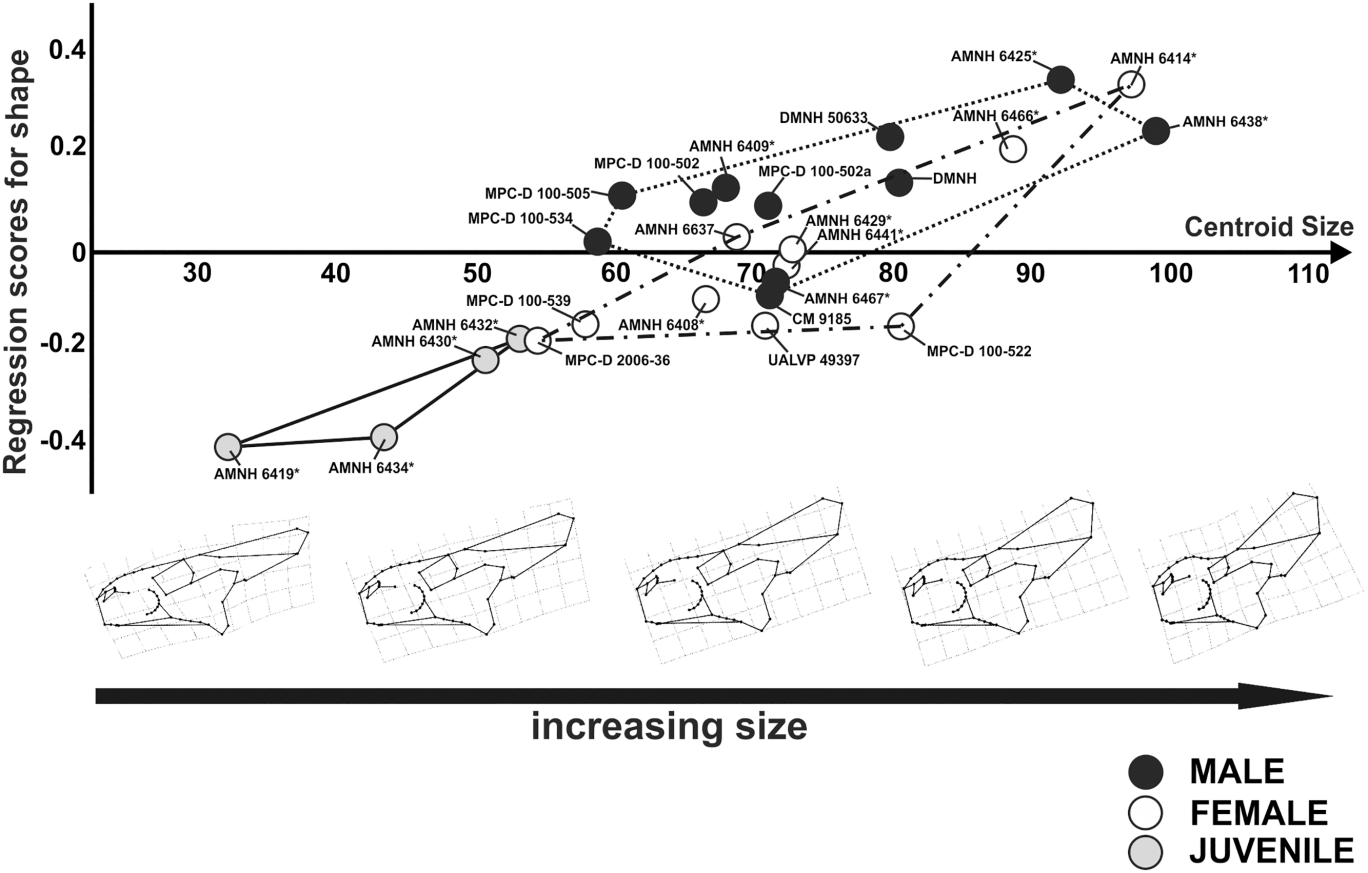
Visualization of shape-size relationship via CCA analysis for cranial shape in lateral view and cranial shape changes associated with the increase of size. Asterisks indicate specimens shared with Dodson [25].

The skulls are characterized by several modifications with the increase of size. The frill develops dorsally, the rostrum gets longer with a developed nasal bump, the external naris tilts upward, the squamosal enlarges caudodorsally and the orbit becomes smaller. Thus, the greatest cranial modifications occur in the frill and in the rostrum during growth (Fig 11).

**Fig 12:**
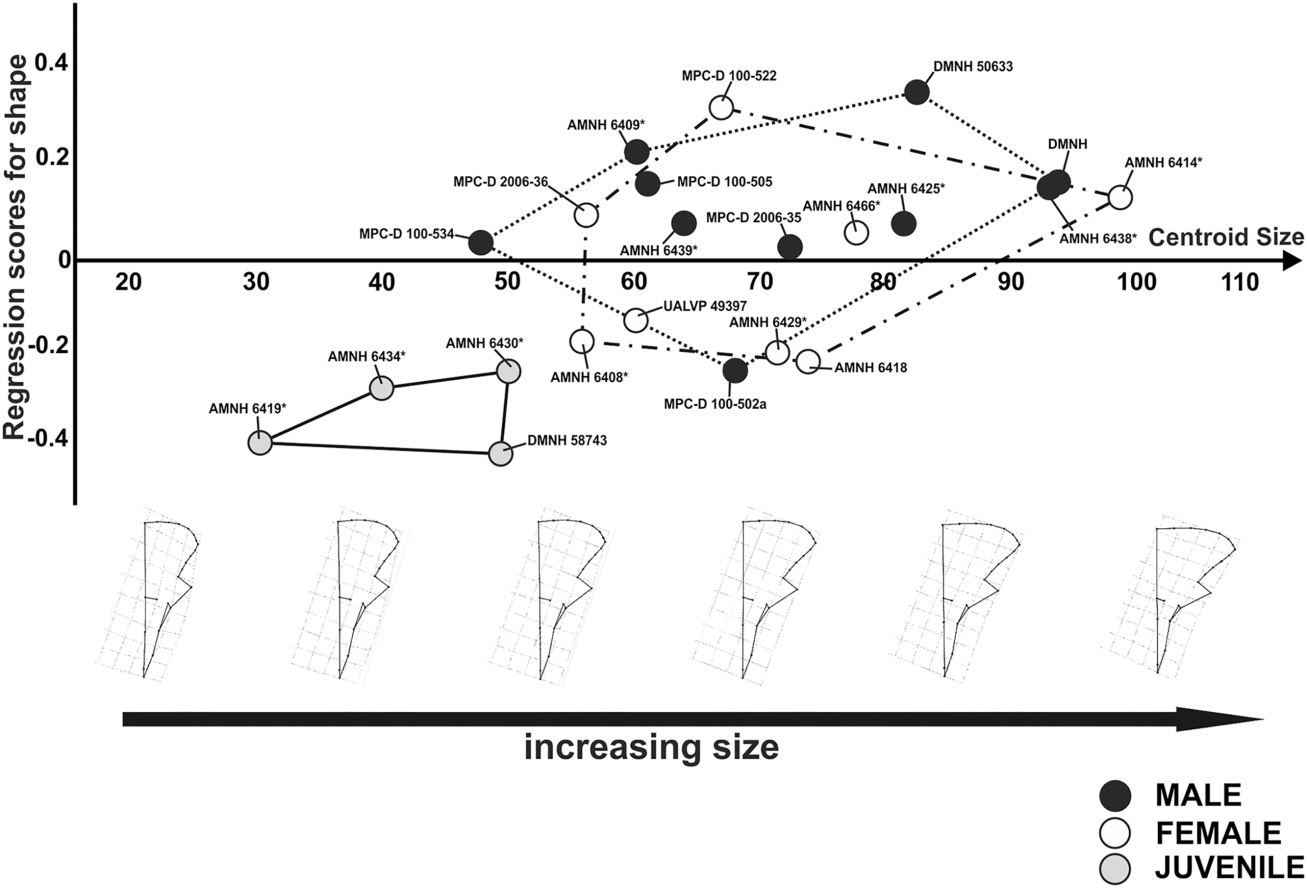
Visualization of shape-size relationship via CCA analysis for cranial shape in dorsal view and relative cranial shape changes associated with the increase of size. Asterisks indicate specimens shared with Dodson [25].

When regressing shape of skull in dorsal view and size, at small size values the skull is juvenile-like. It possesses a short rostrum, a narrow frill and less laterally developed jugal. At large size values, the skull is characterized by a wide frill, laterally developed jugals, and a long rostrum. This morphological variation is adult-like. The major modifications in the dorsal view of skull during growth are the extreme lateral enlargement of the frill and the elongation of the rostrum (Fig 12).

## Discussion

This work is the first to use geometric morphometrics (GM) on a large sample to investigate sexual dimorphism within *P*. *andrewsi*.

As reported previously [23,25,31,39,44], sexual dimorphism within *P*. *andrewsi* should hypothetically be visible in skull shape. Thus, if *P*. *andrewsi* exhibits clear sexual dimorphism, particularly in the frill and in the nasal horn, GM might detect these morphological differences between “males” and “females”. Padian and Horner [3] noted that previous investigations on sexual dimorphism in *P*. *andrewsi* are insufficient to identify sexual dimorphism. The results of this work do not support the hypothesis of sexual dimorphism within *P*. *andrewsi*.

First, considering only linear measurements taken on skulls, no consistent morphological difference appears between “males” and “females” (including adults and sub-adults; Fig 5), as classified under previously used criteria.

Concerning shape, the PCAs performed on the cranial sample, in lateral and dorsal view (Figs 6 and 9, and S1 and S2 Figs), highlighted wide shape variation between juveniles, “males” and “females” and within each group. The hypothetical “males” and “females” have similar, broadly overlapping cranial morphologies. Frill width and nasal height, suggested as critical to distinguish “males” and “females” [25], exhibit a wide range of variation within *P*. *andrewsi* when looking at the whole shape of the skull. Importantly, npMANOVAs (Table 3) do not highlight any differences in shape between hypothetical “males” and “females” within *P*. *andrewsi* as reported above. Similar results are found when testing for size differences or shape differences, for the PC scores, using npANOVA (Table 4).

The sole exception to broad overlap between hypothesized morphotypes concerns rostrum shape (Table 5). Here “males” are significantly morphologically separated from “females”. However this shape separation should be considered quite tentative. Cluster analysis on the rostrum shape (Fig 9C) does not support an evident difference between rostrum morphology of “males” and “females”. For instance, the typical “male” AMNH 6438, as defined in previous works [23,25], resembles the morphology of AMNH 6466, a typical “female”.

Moreover, the morphological changes associated to PC1, that shows significant differences among groups (Table 5), could reveal peramorphic anatomical traits developed at different growth stages.

Overall, previously hypothesized criteria are not suitable to discriminate the two sexes. Thus, we suggest that cranial shape variation previously interpreted as sexual dimorphism is primarily related to ontogenetic shape changes and perhaps also related to intraspecific shape variation (as suggested by Makovicky et al. [73] and Frederickson [74]).

Alternatively, sexual dimorphism may have been present only in the rostrum shape, as highlighted by npANOVA results performed on PC1 values (Table 5), but not evident enough to detect reliably with our current sample size. Of course, additional work and fossil material eventually may reveal evident sexual dimorphism in the skull of *Protoceratops*. However, this will require major efforts to constrain ontogenetic stage (e.g., Makovicky et al. [73]) and a larger, more comprehensive sample. Furthermore, although Dodson’s sex discriminant traits appear unclear, exploring the rostrum and frill morphology individually in lateral view, along with the entire shape of the skull in lateral and dorsal view, shows no evidence of new sex discriminant characters in *Protoceratops*. Additionally, sexual dimorphism in *P*. *andrewsi* does not occur in the cranial shape but may be restricted to the postcranium as recently argued in several works [75,76].

We also must consider the possibility that recognizable sexual dimorphism does occur within our sample, but simply in criteria differing from those of Dodson and others and along sex identifications that differ from those used here. However, we were not able to discern visually any obvious alternative groupings in the sample, either through the PC plots or the cluster analysis of various elements. Some techniques to infer dimorphism statistically have had success in extant and extinct mammals (e.g., [11,77]). However, the techniques are more easily applicable in mammals, which tend to stay at relatively constant size osteologically during adulthood relative to non-avian dinosaurs and which can also be more easily separated into juveniles and adults, removing confounding effects of ontogeny. Given the nature of growth in *Protoceratops*, we do not feel that techniques tested in mammals work here.

Evolutionary change in morphology is another possible explanation for the variation that we see within our *Protoceratops* sample. Unfortunately, stratigraphic data are extremely limited for most specimens, and the hypothesis cannot be tested further.

Exploring the morphological variation of skulls, when regressing shape and size, highlights the broad cranial variation of the frill and rostrum in lateral and dorsal view during ontogeny. From juveniles to adults, the frill and rostrum are characterized by major changes (enlargement of frill and elongation of rostrum) which could be previously misunderstood as distinct morphologies pertaining to two different sexes. However, we cannot exclude non-sexual intraspecific variation within this taxon at different stages of growth.

Missing data or taphonomic distortion potentially are issues with analyses such as ours. Some specimens were excluded from the performed analyses because the fossils were too distorted or damaged. Therefore, some morphological variation within *Protoceratops* was lost because it was not investigated in this work. Nevertheless, the morphospaces still highlight wide morphological disparity within *P*. *andrewsi*. Missing anatomical traits reconstructed using the function fixLMtps() also could affect the results. The estimation of landmarks (conducted to avoid errors during the landmark digitization) is based on the three closest complete specimens in the morphospace and represents a conservative estimation. However, only a few specimens were affected by the reconstruction process, and these only for restricted anatomical regions (e.g., MPC-D 2006.36, AMNH 6441 and MPC-D-100.539). Furthermore, specimens subject to extensive reconstruction, mainly on the parietal, were excluded from the analyses.

Lambert et al. [31] argued for sexual dimorphism within *Protoceratops hellenikorhinus*, following a discriminant function analysis using linear measurements from three skulls of *P*. *hellenikorhinus* and 19 *P*. *andrewsi* specimens already studied by Dodson [25]. Males and females appeared to be distinct here, although one male (AMNH6467) is close to female morphology and juveniles resemble female shape. A recent paper by Mitteroecker and Bookstein [72] argued for the need to have a large sample size to perform those type of analyses to detect morphological differences between groups. Thus, the currently known sample of *P*. *hellenikorhinus* is not sufficient for confident identification of sexual dimorphism. Additionally, if no major sexual dimorphism occurs in *P*. *andrewsi*, it is unlikely that major sexual dimorphism occurred in the skulls of *P*. *hellenikorhinus*.

In the end, even if our results do not strongly support sexual dimorphism within *P*. *andrewsi*, we note that this hypothesis is quite difficult to test using modern statistical methods, particularly for dinosaur fossils. It is probable that males and females occur in the sample, although this distinction does not appear to be reflected in the cranial morphology, and in the frill shape in particular, except for nasal horn shape.

We suggest use of a similar approach for the exploration of the same issue in distinct groups of extinct animals. Collecting a large sample and applying GM allows deep exploration of the sexual-/morphological variation of the cranium or postcranium and assessment of differences in shape between males and females with adequate statistical support. Nonetheless, previous studies [e.g., 78] have suggested that quite large samples are needed to distinguish relatively subtle forms of dimorphism. Ultimate identification of sexual dimorphism in non-avian dinosaurs may indeed be quite difficult.

## Supporting Information

S1 Fig 3D plot of Principal Component Analysis of skulls in lateral view. The black hull represents the “male” morphospace. The red hull represents the “female” morphospace. The green hull represents “juvenile” morphospace. Points dimensions are proportional to specimen Centroid Size.
(PDF)

S2 Fig 3D plot of Principal Component Analysis of skulls in lateral view. The black hull represents the “male” morphospace. The red hull represents the “female” morphospace. The green hull represents “juvenile” morphospace. Points dimensions are proportional to specimen Centroid Size.
(PDF)

S1 File Including the following: 1) Table A. List of material directly photographed for this study and references for those species for which we used published photos or drawings. Bold specimens are shared with those of Dodson [1]. Full references list is appended below. lv = lateral view; dv = dorsal view. 3) Table B. Linear measurements (cm) calculated on skulls of each *Protoceratops andrewsi* specimen occurred in the sample. The numbers of measurements corresponds to those illustrated in Fig 2. Bold specimens are shared with those of Dodson [1]. NA = not available. 4) Table C. Landmark definitions for the four modules (see Fig 4). A, landmark definitions for skull in lateral view. B, landmark definitions for skull in dorsal view. C and D are subunits of skull configuration. Landmarks have identical definitions. 5) Table D. Principal components and eigenvalues for cranial configuration in lateral view. 6) Table E. Principal components and eigenvalues for cranial configuration in dorsal view. 7) Table F. Pair-wise npANOVAs performed on each PCscore variable per-group. Statistically significant results (*p*<0.05) are indicated in bold. Statistically significant results (*p*<0.05) after a Holm correction are shown in the lower left triangle. 8) References.
(PDF)

S2 File Raw Data. TPS files of raw landmark coordinates of the four datasets (skulls in dorsal and lateral view, skulls with the frill excluded and the sole frills).
(ZIP)

## References

[1] Darwin CR. The origin of species by means of natural selection. London: John Murray; 1859.

[2] Darwin CR. The descent of man and selection in relation to sex. London: John Murray; 1871.

[3] Padian K, Horner JR. The evolution of ‘bizarre structures’ in dinosaurs: biomechanics, sexual selection, social selection or species recognition? J Zool. 2011; 283: 3–17. 21552308

[4] Selander RK. Sexual dimorphism and differential niche utilization in birds. Condor. 1966; 68: 113–151.

[5] Berry JF, Shine R. Sexual size dimorphism and sexual selection in turtles (Order Testudines). Oecologia. 1980; 44: 185–191. 28310555 10.1007/BF00572678

[6] Shine R. Ecological causes for the evolution of sexual dimorphism: a review of the evidence. Q Rev Biol. 1989; 64: 419–461. 2697022 10.1086/416458

[7] Chapman RE, Weishampel DB, Hunt G, Rasskin-Gutman D. Sexual dimorphism in dinosaurs. DinoFest Int Proc. 1997: 83–93.

[8] Owens IPF, Hartley IR. Sexual dimorphism in birds: why are there so many different forms of dimorphism. Proc R Soc B. 1998; 265: 397–407.

[9] Mead AJ. Sexual dimorphism and paleoecology in *Teleoceras*, a North American Miocene rhinoceros. Paleobiology. 2000; 26: 689–706.

[10] Badyaev A. Growing apart: an ontogenetic perspective on the evolution of sexual size dimorphism. Trends Ecol Evol. 2002; 17: 369–378.

[11] Van Valkenburgh B, Sacco T. Sexual dimorphism, social behavior, and intrasexual competition in large Pleistocene carnivorans. J Vertebr Paleontol. 2002; 22: 164–169.

[12] Heckert KE, Lucas SG, Heckert AB. The Late Triassic Canjilon quarry (Upper Chinle Groups, New Mexico) phytosaur skulls: evidence of sexual dimorphism in phytosaurs. NM Mus Nat Hist Sci Bull. 2002; 21: 179–188.

[13] Bunce M, Worthy TH, Ford T, Hoppitt W, Willerslev E, Drummond A, et al Extreme reversed sexual size dimorphism in the extinct New Zealand moa *Dinornis* . Nature. 2003; 425: 172–175. 12968178 10.1038/nature01871

[14] Butler MA, Sawyer SA, Losos JB. Sexual dimorphism and adaptive radiation in *Anolis* lizards. Nature. 2007; 447: 202–205. 17495925 10.1038/nature05774

[15] Isles TE. The socio-sexual behaviour of extant archosaurs: implications for understanding dinosaur behavior. Hist Biol. 2009; 21: 139–214.

[16] Ljubisavljević K, Urošević A, Aleksić I, Ivanović A. Sexual dimorphism of skull shape in a lacertid lizard species (*Podarcis* spp., *Dalmatolacerta* sp., *Dinarolacerta* sp.) revealed by geometric morphometrics. Zoology. 2010; 113: 168–174. 10.1016/j.zool.2009.09.003 20439153

[17] Barden HE, Maidment SCR. Evidence for sexual dimorphism in the stegosaur *Kentrosaurus aethiopicus* from the Upper Jurassic of Tanzania. J Vertebr Paleontol. 2011; 31: 641–651.

[18] Foth C, Bona P, Desojo JB. Intraspecific variation in the skull morphology of the black caiman *Melanosuchus niger* (Alligatoridae, Caimaninae). Acta Zool. 2015; 96: 1–13. 25641974

[19] Carpenter K. Eggs, nests, and baby dinosaurs: a look at dinosaur reproduction. Bloomington: Indiana University Press; 2000.

[20] Evans DC, Reisz RR. Anatomy and relationships of *Lambeosaurus magnicristatus*, a crested hadrosaurid dinosaur (Ornithischia) from the Dinosaur Park Formation, Alberta. J Vertebr Paleontol. 2007; 27: 373–393.

[21] Erickson GM, Kristopher Lappin A, Larson P. Androgynous rex—The utility of chevrons for determining the sex of crocodilians and non-avian dinosaurs. Zoology. 2005; 108: 277–286. 16351976 10.1016/j.zool.2005.08.001

[22] Schweitzer MH, Wittmeyer JL, Horner JR. Gender-Specific Reproductive Tissue in Ratites and Tyrannosaurus rex. Science. 2005; 308: 1456–1460. 15933198 10.1126/science.1112158

[23] Brown B, Schlaikjer EM. The structure and relationships of *Protoceratops* . Ann NY Acad Sci. 1940; 40: 133–266.

[24] Farlow JO, Dodson P. The behavioral significance of frill and horn morphology in ceratopsian dinosaurs. Evolution. 1975; 29: 353–361. 28555861 10.1111/j.1558-5646.1975.tb00214.x

[25] Dodson P. Quantitative aspects of relative growth and sexual dimorphism in *Protoceratops* . J Paleontol. 1976; 50: 929–940.

[26] Dodson P. The horned dinosaurs. Princeton: Princeton University Press; 1996.

[27] Lehman TM. The ceratopsian subfamily Chasmosaurinae: sexual dimorphism and systematics In: Carpenter K, Currie PJ, editors. Dinosaur Systematics: Approaches and Perspectives. Cambridge: Cambridge University Press; 1990 pp. 211–230.

[28] Lehman TM. Growth and population age structure in the horned dinosaur *Chasmosaurus* In: Carpenter K, editor. Horns and Beaks. Bloomington: Indiana University Press; 2007 pp. 259–317.

[29] Sampson SD. Sex and destiny: the role of mating signals in speciation and macroevolution. Hist Biol. 1999; 13: 173–197.

[30] Sampson SD. Speculations on the socioecology of ceratopsid dinosaurs (Ornithischia: Neoceratopsia) In: Tanke D, Carpenter K, editors. Mesozoic vertebrate life. Bloomington: Indiana University Press; 2001 pp. 263–276.

[31] Lambert O, Godefroit P, Li H, Shang CY, Dong ZM. A new species of *Protoceratops* (Dinosauria, Neoceratopsia) from the Late Cretaceous of Inner Mongolia (P. R. China). Bull Inst R Sc N B-S. 2001; 71(suppl.): 5–28.

[32] Ryan MJ, Russell AP, Eberth DE, Currie PJ. The taphonomy of a *Centrosaurus* (Ornithischia: Ceratopsidae) bone bed from the Dinosaur Park Formation (Upper Campanian), Alberta, Canada, with comments on cranial ontogeny. Palaios. 2001; 16: 482–506.

[33] Dodson P, Forster CA, Sampson SD. Ceratopsidae In: Weishampel DB, Dodson P, Osmolska H, editors. The Dinosauria. 2nd ed Berkeley: University of California Press; 2004 pp 494–513.

[34] Mallon JC, Holmes RB. A reevaluation of sexual dimorphism in the postcranium of the chasmosaurine ceratopsid *Chasmosaurus belli* (Dinosauria: Ornithischia). Can Field Nat. 2006; 120: 403–412.

[35] Handa N, Watabe M, Tsogtbaatar K. New specimens of *Protoceratops* (Dinosauria: Neoceratopsia) from the Upper Cretaceous in Udyn Sayr, southern Gobi area, Mongolia. Paleontol Res, 2012; 16: 179–198.

[36] Happ JW, Morrow CM. Separation of *Triceratops* (Dinosauria: Ceratopsidae) into two allopatric species by cranial morphology: J Vertebr Paleontol, 1996; 16: 40A.

[37] Dodson P. On the status of the ceratopsids *Monoclonius* and *Centrosaurus* In: Carpenter K, Currie PJ, editors. Dinosaur Systematics: Approaches and Perspectives. Cambridge: Cambridge University Press; 1990 pp 231–243.

[38] Chapman RE. Shape analysis in the study of dinosaur morphology In: Carpenter K, Currie PJ, editors. Dinosaur Systematics: Approaches and Perspectives. Cambridge: Cambridge University Press; 1990 pp 21–42.

[39] Gregory WK, Mook CC. On *Protoceratops*, a primitive ceratopsian dinosaur from the Lower Cretaceous of Mongolia. Am Mus Novit. 1925; 156: 1–9.

[40] Kurzanov SM. Sexual dimorphism in protoceratopsians. Palaeont J. 1972; 1: 91–97. 4340655

[41] Sampson SD, Ryan MJ, Tanke DH. Craniofacial ontogeny in centrosaurines dinosaurs (Ornithischia: Ceratopsidae): Taxonomic and behavioral implications. Zool J Linn Soc. 1997; 121: 293–337.

[42] Horner JR, Goodwin MB. Major cranial changes during *Triceratops* ontogeny. Proc R Soc B. 2006; 273: 2757–2761. 17015322 10.1098/rspb.2006.3643 PMC1635501

[43] Frederickson JA, Tumarkin-Deratzian AR. Craniofacial ontogeny in *Centrosaurus apertus* . PeerJ. 2014; 10.7717/peerj.252 PMC3933270 24688836

[44] Granger W, Gregory WK. *Protoceratops andrewsi*, a pre-ceratopsian dinosaur from Mongolia. Am Mus Novit. 1923; 72: 1–9.

[45] Rohlf FJ, Slice DE. Extensions of the Procrustes method for the optimal superimposition of landmarks. Syst Zool. 1990; 39: 40–59.

[46] Dodson P. Comparative craniology of the Ceratopsia. Am J Sci. 1993; 293A: 200–234.

[47] Rohlf FJ, Marcus LF. A revolution in morphometrics. Trends Ecol Evol. 1993; 8: 129–132. 10.1016/0169-5347(93)90024-J 21236128

[48] Adams DC, Rohlf FJ, Slice DE. Geometric morphometrics: ten years of progress following the ‘revolution’. Ital J Zool. 2004; 71: 5–16.

[49] Mitteroecker P, Gunz P, Bookstein FL. Heterochrony and geometric morphometrics: a comparison of cranial growth in *Pan paniscus* versus *Pan troglodytes* . Evol Dev. 2005; 7: 244–258. 15876197 10.1111/j.1525-142X.2005.05027.x

[50] Adams DC, Collyer ML. A general framework for the analysis of phenotypic trajectories in evolutionary studies. Evolution. 2009; 63: 1143–1154. 10.1111/j.1558-5646.2009.00649.x 19210539

[51] Mitteroecker P, Gunz P. Advances in geometric morphometrics. Evol Biol. 2009; 36: 235–247.

[52] Adams DC, Nistri A. Ontogenetic convergence and evolution of foot morphology in European cave salamanders (Family: Plethodontidae). BMC Evol Biol. 2010; 10: 1–10. 10.1186/1471-2148-10-1 20637087 PMC2927916

[53] Piras P, Maiorino L, Raia P, Marcolini F, Salvi D, Vignoli L, et al Functional and phylogenetic constraints in Rhinocerotinae craniodental morphology. Evol Ecol Res. 2010; 12: 897–928.

[54] Piras P, Maiorino L, Teresi L, Meloro C, Lucci F, Kotsakis T, et al Bite of the cats: relationships between functional integration and mechanical performance as revealed by mandible geometry. Syst Biol. 2013; 62: 878–900. 10.1093/sysbio/syt053 23925509

[55] Prevosti FJ, Turazzini GF, Chemisquy MA. Morfología craneana en tigres dientes de sable: alometría, functión y filogenia. Ameghiniana. 2010; 47: 239–256.

[56] Young MT, Brusatte SL, Ruta M, De Andrade MB. The evolution of Metriorhynchoidea (Mesoeucrocodylia, Thalattosuchia): an integrated approach using geometric morphometrics, analysis of disparity, and biomechanics. Zool J Linn Soc. 2010; 158: 801–859.

[57] Sicuro FL. Evolutionary trends on extant cat skull morphology (Carnivora: Felidae): a three-dimensional geometrical approach. Biol J Linn Soc. 2011; 103: 176–190.

[58] Figueirido B, MacLeod N, Krieger J, De Renzi M, Pérez-Claros JA, Palmqvist P. Constraint and adaptation in the evolution of carnivoran skull shape. Paleobiology. 2011; 37: 490–518.

[59] Baab KL, McNulty KP, Rohlf FJ. The shape of human evolution: a geometric morphometrics perspective. Evol Anthropol. 2012; 21: 151–165. 10.1002/evan.21320 22907868

[60] Zelditch ML, Swiderski DL, Sheets HD. Geometric morphometrics for biologists: a primer. 2nd ed Amsterdam: Elsevier Academic Press; 2012

[61] Foth C, Rauhut OWM. Macroevolutionary and morphofunctional patterns in theropod skulls: A morphometric approach. Acta Palaeontol Pol. 2013; 58: 1–16.

[62] Marcus LF, Hingst-Zaher E, Zaher H. Application of landmarks morphometrics to skull representing the orders of living mammals. Hystrix. 2000; 11: 27–47.

[63] Mullin SK, Taylor PJ. The effects of parallax on geometric morphometric data. Comput Biol Med. 2002; 32: 455 464. 12356495 10.1016/s0010-4825(02)00037-9

[64] Rohlf FJ. TpsDig2. 2.16 ed 2013 Stony Brook, N.Y: Published by the Author.

[65] Schlager S. Morpho: Calculations and visualizations related to Geometric Morphometrics. R package v0.23.3. 2013. http://CRAN.R-project.org/package=Morpho.

[66] Maiorino L, Farke AA, Kotsakis T, Piras P. Is *Torosaurus Triceratops*? Geometric morphometric evidence of Late Maastrichtian ceratopsid dinosaurs. PLOS ONE. 2013; 10.1371/journal.pone.0081608 PMC3841114 24303058

[67] Bookstein FL, Streissguth AP, Sampson PD, Connor PD, Barr HH. Corpus callosum shape and neuropsychological deficits in adult males with heavy fetal alcohol exposure. NeuroImage. 2002; 15: 233–251. 11771992 10.1006/nimg.2001.0977

[68] Perez SI., Bernal V, Gonzalez PN. Differences between sliding semi-landmark methods in geometric morphometrics, with an application to human craniofacial and dental variation. J Anat. 2006; 208: 769–784. 16761977 10.1111/j.1469-7580.2006.00576.x PMC2100233

[69] Bookstein FL. Morphometric Tools for Landmark Data: Geometry and Biology. Cambridge: Cambridge University Press; 1991.

[70] Bookstein FL. Size and shape spaces for landmark data in two dimensions. Stat Sci. 1986; 1: 181–242.

[71] Oksanen JF, Blanchet G, Kindt R, Legendre P, Minchin PR, O’Hara RB, et al. Vegan: Community Ecology Package. R package v2.0–2. 2011. Available at http://CRAN.R-project.org/package=vegan.

[72] Mitteroecker P, Bookstein F. Linear discrimination, ordination, and the visualization of selection gradients in modern morphometrics. Evol Biol. 2011; 38: 100–114.

[73] Makovicky P, Sadleir R, Dodson P, Erickson G, Norell M. Life history of *Protoceratops andrewsi* from Bayn Zag, Mongolia. J Vertebr Paleontol. 2007; 27: 109A.

[74] Frederickson J. Sexual dimorphism is a derived condition in the evolution of horned dinosaurs (Ornithischia, Neoceratopsia): evidence from growth series of Pachyrhinosaurus lakustai and Protoceratops andrewsi. Geol S Am. 2011; 43S: 119.

[75] Tereschenko VS. Sexual Dimorphism in the Postcranial Skeleton of Protoceratopids (Neoceratopsia, Protoceratopsidae) from Mongolia. Paleontol J. 2001; 4: 79–89.

[76] Tereschenko VS. Adaptive Features of Protoceratopoids (Ornithischia: Neoceratopsia). Paleontol J. 2008; 42: 273–286. 10.2345/0899-8205(2008)42[273:OOOCOA]2.0.CO;2 18662057

[77] Rehg JA, Leigh SR. Estimating Sexual Dimorphism and Size Differences in the Fossil Record: A Test of Methods. Am J Phys Anthropol. 1999; 110: 95–104. 10490471 10.1002/(SICI)1096-8644(199909)110:1<95::AID-AJPA8>3.0.CO;2-J

[78] Prieto-Marquez A, Gignac PM, Joshi S. Neontological evaluation of pelvic skeletal attributes purported to reflect sex in extinct non-avian archosaurs. J Vertebr Paleontol. 2007; 27: 603–609.

[79] Mallon JC, Holmes R, Eberth DA, Ryan MJ, Anderson JS. Variation in the skull of *Anchiceratops* (Dinosauria, Ceratopsidae) from the Horseshoe Canyon Formation (Upper Cretaceous) of Alberta. J Vertebr Paleontol. 2011; 31: 1047–1071.

