# Supplementary material for: Males Resemble Females: Re-Evaluating Sexual Dimorphism in Protoceratops andrewsi (Neoceratopsia, Protoceratopsidae)
Source: PLoS One. 2015 May 7;10(5):e0126464. doi: 10.1371/journal.pone.0126464 (PMC4423778; doi:10.1371/journal.pone.0126464)
Supplement: S1 Fig — The black hull represents the “male” morphospace. The red hull represents the “female” morphospace. The green hull represents “juvenile” morphospace. Points dimensions are proportional to specimen Centroid Size. (PDF) [file pone.0126464.s001.pdf]

Figure S2. 3D plot of Principal Component Analysis of skulls in lateral view. The black hull represents the "male" morphospace. The red hull represents the "female" morphospace. The green hull represents "juvenile" morphospace. Points dimensions are proportional to specimen Centroid Size.
