# Supplementary material for: Males Resemble Females: Re-Evaluating Sexual Dimorphism in Protoceratops andrewsi (Neoceratopsia, Protoceratopsidae)
Source: PLoS One. 2015 May 7;10(5):e0126464. doi: 10.1371/journal.pone.0126464 (PMC4423778; doi:10.1371/journal.pone.0126464)
Supplement: S1 File — Including the following: 1) Table A. List of material directly photographed for this study and references for those species for which we used published photos or drawings. Bold specimens are shared with those of Dodson [1]. Full references list is appended below. lv = lateral view; dv = dorsal view. 3) Table B. Linear measurements (cm) calculated on skulls of each Protoceratops andrewsi specimen occurred in the sample. The numbers of measurements corresponds to those illustrated in Fig 2. Bold specimens are shared with those of Dodson [1]. NA = not available. 4) Table C. Landmark definitions for the four modules (see Fig 4). A, landmark definitions for skull in lateral view. B, landmark definitions for skull in dorsal view. C and D are subunits of skull configuration. Landmarks have identical definitions. 5) Table D. Principal components and eigenvalues for cranial configuration in lateral view. 6) Table E. Principal components and eigenvalues for cranial configuration in dorsal view. 7) Table F. Pair-wise npANOVAs performed on each PCscore variable per-group. Statistically significant results (p<0.05) are indicated in bold. Statistically significant results (p<0.05) after a Holm correction are shown in the lower left triangle. 8) References. (PDF) [file pone.0126464.s003.pdf]

Table A. List of material directly photographed for this study, references for those species for which we used published photos or drawings. Bold specimens are shared with those of Dodson [1]. Full references list is appended below. lv = lateral view; dv = dorsal view.

COLLECTION NUMBER	MATERIAL	Basal Skull Length (cm)	GROWTH STAGE	SEX	REFERENCES
**AMNH 6408**	Skull (lv-dv)	23.5	sub-adult	Female	
**AMNH 6419**	Skull (lv-dv)	7.6	juvenile	---	Brown and Schlaikjer [2]
**AMNH 6432**	Skull (lv-dv)	16.8	juvenile	---	
**AMNH 6434**	Skull (lv-dv)	12.2	juvenile	---	
**AMNH 6466**	Skull (lv-dv)	37.7	adult	Female	
**AMNH 6438**	Skull (lv-dv)	35.2	adult	Male	
**AMNH 6439**	Skull (dv)	27.1	sub-adult	Male	
**AMNH 6409**	Skull (lv-dv)	19.1	sub-adult	Male	
**AMNH 6414**	Skull (lv-dv)	34.1	adult	Female	
AMNH 6418	Skull (lv-dv)	22.7	sub-adult	Female	
**AMNH 6425**	Skull (lv-dv)	31.3	adult	Male	
**AMNH 6429**	Skull (lv-dv)	26.9	sub-adult	Female	
**AMNH 6430**	Skull (lv-dv)	13.7	juvenile	---	
**AMNH 6441**	Skull (lv)	27.2	adult	Female	
**AMNH 6467**	Skull (lv)	26	adult	Male	
AMNH 6637	Skull (lv-dv)	22.3	sub-adult	Female	
UALVP 49397 (cast)	Skull (lv-dv)	22.4	adult	Female	
CM 9185	Skull (lv)	25	adult	Male	
DMNH no code (cast)	Skull (lv-dv)	26.7	adult	Male	
DMNH 50633 (cast)	Skull (lv-dv)	27.4	adult	Male	
DMNH 58743 (cast)	Skull (dv)	15.5	juvenile	---	
MPC-D 100-522	Skull (lv-dv)	27.4	adult	Female	
MPC-D 100-502	Skull (lv)	20.8	adult	Male	
MPC-D 100-502a	Skull (lv-dv)	22.6	adult	Male	
MPC-D 2006.36	Skull (lv-dv)	16.5	sub-adult	Female	
MPC-D 2006.35	Skull (dv)	17.8	sub-adult	Male	
MPC-D 100-505	Skull (lv-dv)	17.1	sub-adult	Male	
MPC-D 100-534	Skull (lv-dv)	17.4	sub-adult	Male	Handa et al. [3]
MPC-D 100-539	Skull (lv-dv)	19.2	sub-adult	Female	Handa et al. [3]

Table B. Linear measurements (cm) calculated on skulls of each *Protoceratops andrewsi* specimen occurred in the sample. The numbers of measurements corresponds to those illustrated in Fig. 2. Bold specimens are shared with those of Dodson [1]. NA = not available.

LABELS	1. Width of the Frill	2. Postorbital width of the Skull	3. Length of the Frill	4. Width of skull across jugals	5. Nasal height of Skull	6. Height of the Frill	7. Length of external nares	8. Width of external nares
**AMNH 6467**	NA	NA	NA	NA	14.1	21.7	4.7	2
DMNH 50633	67.36	15.72	27.62	40.2	15.92	28.8	5.1	1.85
DMNH no code	66.76	18.86	35.26	41.2	16.8	29	5.2	1.7
**AMNH 6409**	41.56	10.87	17.15	30.48	11.7	26.3	3.8	1.3
AMNH 6418	33.18	12.53	26.27	23.46	NA	16.81	NA	NA
**AMNH 6425**	53.14	15.35	32.73	35.94	19	33.1	5.25	1.89
**AMNH 6429**	34.21	15.07	24	34.2	11.8	23.4	3.82	1.36
**AMNH 6430**	21.36	7.3	15.04	15.68	6.18	11.1	1.88	0.8
**AMNH 6441**	NA	NA	NA	NA	11.05	20.5	3.93	1.64
AMNH 6637	NA	10.4	NA	22.86	11.2	23.61	2.9	1.27
UALVP 49397	26.84	9.83	21.57	18.82	11.42	20.52	3.27	1.26
CM 9185	37.32	13.4	NA	NA	12.43	23.1	3.86	1.95
MPC-D 100.502	38.6	12.9	23.25	24.64	12.8	20	3.3	1.29
MPC-D 100.502a	30.2	12.4	24.05	27.46	13.2	24.6	3.37	1.24
MPC-D 100.505	38.03	11.6	20.32	21.97	10.92	20.14	2.9	1
MPC-D 2006.36	33.02	12.2	16.6	26.36	8.04	13.16	2.75	1.3
**AMNH 6408**	25.1	9.85	15	23.34	10.95	18.1	3.73	1.61
**AMNH 6432**	21.13	9.8	13.96	19.74	7.62	13.8	2.47	1.2

**AMNH 6434**	11.92	5.63	9.63	11.4	4.8	8.8	2.03	0.84
**AMNH 6438**	62.38	19.22	30.64	50.76	25	39.3	8.49	2.9
**AMNH 6466**	47.49	15.69	21.93	43.64	17.02	29.6	6.31	2.6
**AMNH 6419**	7.6	4.3	5.44	7.27	3.1	5.4	0.83	0.31
MPC-D 100 534	27.91	8.8	16.88	19	10.64	17.6	2.9	1.19
MPC-D 100 539	27.72	10	16.8	19.18	10.04	15	2.76	1.46
DMNH 58743	17.92	8.5	13.6	17	NA	NA	NA	NA
**AMNH 6413**	60.25	18.66	29.34	38.63	NA	NA	NA	NA
**AMNH 6414**	51	16.48	25.46	36.37	18.1	34	6	1.96
**AMNH 6439**	43.5	13.67	21.65	33	NA	NA	NA	NA
MPC D 100.522	46.67	13.9	22	34.32	12.79	23.82	3.87	1.97
MPC D 2006.35	43.63	12.96	27.56	34.51	NA	NA	NA	NA

Table C. Landmark definitions for the four modules (see Fig. 4). A, landmark definitions for skull in lateral view. **B,** landmark definitions for skull in dorsal view. **C** and **D** are subunits of skull configuration. Landmarks have identical definitions.

**A, landmark definitions for skull in lateral view**	
**Landmark #**	**Anatomical definition**
1	upper contact of premaxilla–rostral
2	upper contact of premaxilla–nasal
3	maximum curvature point of naris at ventral edge
4	lower contact of premaxilla–nasal
5	maximum curvature point of narial opening at caudo–dorsal edge
6	caudal contact of nasal–premaxilla
7	upper contact of maxilla–premaxilla
8	dorsal tip of antorbital fenestra
9	caudal tip of antorbital fenestra
10	ventral tip of antorbital fenestra
11	lower contact of premaxilla–rostral
12	lower tip of premaxilla
13	lower contact of premaxilla–maxilla
14	maximum curvature point of jugal
15	intersection of jugal–alveolar process of maxilla
16	epijugal tip
17	lower tip of quadrate
18	contact of jugal–quadratojugal
19	ventral tip of infratemporal process
20	maximum curvature point of infratemporal fenestra
21	contact of quadrate–squamosal
22	lower tip of squamosal
23	parieto–squamosal contact
24	maximum curvature point of parietal
25	dorsal tip of parietal midline
26	rostral tip of supratemporal fenestra
27	dorsal contact of postorbital–squamosal

28	contact of jugal–squamosal
29	dorsal tip of the orbit
30	caudal tip of the orbit
31	jugal–postorbital contact at the orbit rim
32	ventral tip of the orbit
33	rostral tip of the orbit
34	projection of LM 33 on the nasal edge
**B, landmark definitions for skull in dorsal view**	
1	caudal tip of midline bar on the parietal edge
2	parietal–squamosal contact
3	external tip of squamosal blade
4	intersection between squamosal and quadrate
5	epijugal tip
6	contact of frontal–parietal
7	rostral tip of supratemporal fenestra
8	caudal tip of the orbit
9	jugal–postorbital contact
10	rostral tip of the orbit
11	contact of premaxilla–maxilla
12	contact of nasal–frontal
13	contact of rostral–premaxilla
14	rostral tip of the rostral

Table D. Principal Components and Eigenvalues for cranial configuration in lateral view**.**

Principal Component #	Eigenvalues	Proportion of Variance	Cumulative Proportion of Variance
PC1	4.620829e-03	34.74727%	34.74727%
PC2	1.699811e-03	12.78208%	47.52936%
PC3	1.406206e-03	10.57425%	58.10361%
PC4	9.164055e-04	6.89109%	64.99471%
PC5	8.696161e-04	6.53925%	71.53397%
PC6	5.931191e-04	4.46008%	75.99405%
PC7	4.933990e-04	3.71021%	79.70426%
PC8	4.211677e-04	3.16705%	82.87132%
PC9	4.125808e-04	3.10248%	85.97381%
PC10	2.742485e-04	2.06226%	88.03607%
PC11	2.650658e-04	1.99321%	90.02929%
PC12	2.135439e-04	1.60578%	91.63508%
PC13	2.016648e-04	1.51645%	93.15154%
PC14	1.708122e-04	1.28445%	94.43600%
PC15	1.640000e-04	1.23323%	95.66923%

Table E. Principal Components and Eigenvalues for cranial configuration in dorsal view**.**

Principal Component #	Eigenvalues	Proportion of Variance	Cumulative Proportion of Variance
PC1	6.071796e-03	41.36824%	41.36824%
PC2	4.483050e-03	30.54382%	71.91207%
PC3	1.526533e-03	10.40054%	82.31261%
PC4	8.363317e-04	5.69807%	88.01069%
PC5	4.609563e-04	3.14057%	91.15127%
PC6	2.981580e-04	2.03140%	93.18267%
PC7	2.659052e-04	1.81165%	94.99433%
PC8	1.819143e-04	1.23941%	96.23375%

Table F. Pair-wise nonparametric permuted ANOVA performed on each PCscore variable per-group. Statistically significant results ($p<0.05$) are indicated in bold. Statistically significant results ($p<0.05$) after a Holm correction are shown in the lower left triangle.

	Male	Female	Juvenile
Skull (lateral view) – PC1(34.7%)			
Male (n= 11)	---	0.068	**0.001**
Female (n= 10)	0.061	---	**0.03**
Juvenile (n= 4)	**0.033**	0.058	---
Skull (lateral view) – PC2(12.7%)			
Male (n= 11)	---	0.26	**0.002**
Female (n= 10)	0.26	---	0.11
Juvenile (n= 4)	**0.007**	0.23	---
Skull (lateral view) – PC3(10.5%)			
Male (n= 11)	---	0.33	0.56
Female (n= 10)	0.66	---	**0.08**
Juvenile (n= 4)	0.66	0.23	---
Skull (lateral view) – PC4(6.8%)			
Male (n= 11)	---	0.3	0.99
Female (n= 10)	0.95	---	0.46
Juvenile (n= 4)	0.99	0.95	---
Skull (lateral view) – PC5(6.5%)			
Male (n= 11)	---	0.33	0.8
Female (n= 10)	0.78	---	0.25
Juvenile (n= 4)	0.8	0.78	---
Skull (lateral view) – PC6(4.4%)			
Male (n= 11)	---	0.63	0.9
Female (n= 10)	1	---	0.51
Juvenile (n= 4)	1	1	---
Frill (lateral view) – PC1(32.05%)			
Male (n= 11)	---	0.35	**0.023**
Female (n= 11)	0.108	---	**0.025**
Juvenile (n= 4)	**0.008**	**0.027**	---
Frill (lateral view) – PC2(21.04%)			
Male (n= 11)	---	0.056	**0.011**
Female (n= 11)	0.11	---	0.51
Juvenile (n= 4)	**0.03**	0.51	---
Frill (lateral view) – PC3(12.80%)			
Male (n= 11)	---	0.12	0.52
Female (n= 11)	0.36	---	0.2
Juvenile (n= 4)	0.51	0.42	---
Frill (lateral view) – PC4(10.22%)			
Male (n= 11)	---	0.4	0.09
Female (n= 11)	0.38	---	**0.03**
Juvenile (n= 4)	0.19	0.11	---

Frill (lateral view) – PC5(6.34%)			
Male (n= 11)	---	0.94	0.11
Female (n= 11)	0.94	---	0.18
Juvenile (n= 4)	0.31	0.35	---
Frill (lateral view) – PC6(4.82%)			
Male (n= 11)	---	0.74	0.57
Female (n= 11)	1	---	0.73
Juvenile (n= 4)	1	1	---
Skull (dorsal view) – PC1(39.92%)			
Male (n= 10)	---	0.17	**0.011**
Female (n= 8)	0.18	---	**0.005**
Juvenile (n= 4)	**0.004**	**0.02**	---
Skull (dorsal view) – PC2(29.5%)			
Male (n= 10)	---	0.22	0.38
Female (n= 8)	0.66	---	0.87
Juvenile (n= 4)	0.79	0.87	---
Skull (dorsal view) – PC3(12.14%)			
Male (n= 10)	---	0.43	0.69
Female (n= 8)	1	---	0.43
Juvenile (n= 4)	1	1	---
Skull (dorsal view) – PC4(5.58%)			
Male (n= 10)	---	0.32	0.65
Female (n= 8)	0.97	---	0.74
Juvenile (n= 4)	1	1	---

References

1. Dodson P (1976) Quantitative aspects of relative growth and sexual dimorphism in *Protoceratops*. J Paleontol 50: 929–940.

2. Brown B, Schlaikjer EM (1940) The structure and relationships of *Protoceratops*. Ann NY Acad Sci 40: 133–266.

3. Handa N, Watabe M, Tsogtbaatar K (2012) New specimens of *Protoceratops* (Dinosauria: Neoceratopsia) from the Upper Cretaceous in Udyn Sayr, southern Gobi area, Mongolia. Paleontol Res 16: 179–198.
